# Analysis of the Global Disease Burden of Down Syndrome Using YLDs, YLLs, and DALYs Based on the Global Burden of Disease 2019 Data

**DOI:** 10.3389/fped.2022.882722

**Published:** 2022-04-29

**Authors:** Qingting Bu, Rong Qiang, Hua Cheng, Anmin Wang, Huangtao Chen, Zhenyu Pan

**Affiliations:** ^1^Department of Genetics, Northwest Women's and Children's Hospital, Xi'an, China; ^2^Department of Pharmacy, Xi'an Children's Hospital, Xi'an, China; ^3^Department of Neurosurgery, The Second Affiliated Hospital of Xi'an Jiaotong University, Xi'an, China; ^4^School of Public Health, Xi'an Jiaotong University Health Science Center, Xi'an, China

**Keywords:** Down syndrome, global disease burden, trend, YLD, YLL, DALY

## Abstract

**Purpose:**

This study aimed to determine Down syndrome (DS) burden using years lived with disability (YLDs), years of life lost (YLLs), disability-adjusted life years (DALYs), and the trends in these parameters.

**Methods:**

We obtained the annual YLDs, YLLs, DALYs, and age-standardized rates (ASRs) of DS from 2010 to 2019 using the Global Health Data Exchange tool. The estimated annual percentage changes (EAPCs) in ASR were used to quantify and evaluate DS burden trends. Gaussian-process regression and Pearson's correlation coefficient were used to assess the relationship between DS burden and socio-demographic index (SDI).

**Results:**

Global DALYs decreased by 2.68% from 2010 to 2019 but the ASR was stable, which was mostly explained by the stability in the ASR for YLLs. The ASR of YLDs showed an increasing trend (EAPC = 1.07, 95% CI = 0.45 to 1.69). There was notable regional imbalance, with most of the DALYs or ASRs in areas with relatively low SDI. The DALY rates of DS were mostly from the YLLs of children younger than 1 year. Lower SDI areas tended to have higher DS burdens (ρ = −0.3, *p* < 0.001).

**Conclusion:**

This systematic analysis of the global disease burden of DS from 2010 to 2019 revealed that although the global DS DALY and YLL rate is stable, the YLD rate is increasing. And the DS burden varies significantly differences among regions or countries. The present results suggest that future strategies should focus on DS-related deaths in children younger than 1 year and the DS burden in low-SDI regions or countries, since this may be effective in further reducing DS burden.

## Introduction

Down syndrome (DS) is a chromosomal abnormality consisting of the presence of a third copy of chromosome 21 in somatic cells due to nondisjunction (1). DS is the most-common chromosomal condition associated with intellectual disability and is characterized by various additional clinical manifestations. It occurs in ~1 of 800 births worldwide (2). In the United States, DS occurs in approximately 5,000 live births annually, and more than 200,000 people are currently living with this condition (3). Children with DS have multiple malformations, medical conditions, and cognitive impairment (4, 5).

The DS phenotype varies, but multiple features typically enable the experienced clinician to consider a diagnosis. The more-common physical findings include hypotonia, small brachycephalic head, epicanthal folds, flat nasal bridge, upward-slanting palpebral fissures, Brushfield spots, small mouth, small ears, excessive skin at the nape of the neck, a single transverse palmar crease, and a short fifth finger with clinodactyly and wide spacing, often also with a deep plantar groove between the first and second toes. The degree of cognitive impairment typically varies from mild (IQ = 50–70) to moderate (IQ = 35–50), but is occasionally severe (IQ = 20–35). There are significant risks of hearing loss (75%), obstructive sleep apnea (50–79%), otitis media (50–70%), eye disease (60%) including cataracts (15%) and severe refractive errors (50%), and congenital heart defects (50%), while other comorbidities include neurological dysfunction (1–13%), gastrointestinal atresia (12%), hip dislocation (6%), thyroid disease (4–18%), transient myeloproliferative disorder (4–10%), leukemia (1%), and Hirschsprung's disease (1%) (6). Among fetuses with DS, 27% of pregnancies are reportedly terminated, 4% are spontaneous late abortions or stillbirths, and 12% die within 1 year of birth (7). There are reports from various countries that the number of fetuses conceived with DS has increased concomitantly with the mean age of pregnant females (8–12). DS represents a significant burden on affected patients and their families, as well as society as a whole. However, no previous study has systematically assessed the global disease burden of DS.

The present study therefore aimed to determine the global disease burden of DS using years lived with disability (YLDs), years of life lost (YLLs), and disability-adjusted life years (DALYs) from different periods, regions, and populations based on the Global Burden of Disease (GBD) 2019 data. The findings of this study can be useful when designing more-effective region-specific policies and methods for improving the disease burden of DS.

## Methods

### Data Source

Data sources from the GBD 2019 data can be explored using the online Global Health Data Exchange (GHDx) data source tool (http://ghdx.healthdata.org/gbd-results-tool). The GHDx is a data catalog created and supported by the Institute for Health Metrics and Evaluation, an independent global health research center at the University of Washington. The GBD 2019 is the most comprehensive multi-institutional and multi-individual global collaborative epidemiological database for estimating the annual burden of 369 diseases and injuries (e.g., incidence, prevalence, mortality, YLDs, YLLs, and DALYs) for 204 countries and territories according to sex and age group from 1990 to 2019 (13–15). YLDs refer to the number of years lived with any short-term or long-term health loss weighted by the severity of the disability, YLLs refer to how many years of life were lost due to premature mortality, and DALYs were calculated by summing YLLs and YLDs. The GBD case definition of DS includes the International Classification of Diseases codes Q90.0–Q90.2, and Q90.9. In this study, we obtained data from the GBD 2019 including YLDs, YLLs and DALYs of DS, their rates, their age-standardized rates (ASRs) from 2010 to 2019, the change between 2010 and 2019 and 95% uncertainty interval (UI) of the estimates according to sex, age, region, and country. In GBD, every estimate is calculated 1,000 times, sampled from the distribution each time, and sorted from the minimum value to the maximum value. The 95% UI is determined by the 25th and 975th values of 1,000 values. Many types of data sources are used in the estimation of DS, including literature prevalence, with-condition mortality and excess mortality data, birth prevalence and neonatal with-condition mortality data from a number of international birth defects registries and surveillance systems, etc. The estimates of DS are mostly based on modeling when the good data sources are lacking, which may cause some biases. More detailed information on the GBD 2019 data, standardization methods and its limitations is available elsewhere (15). The socio-demographic index (SDI) has been used to comprehensively evaluate the social development levels within countries and regions. This metric is a geometric average ranging from 0 to 1 in each country or region calculated by combining the total fertility rate of females younger than 25 years, the education level of people aged at least 15 years, and the logarithm of the per-capita income distribution. Lower SDI values indicate lower social development levels, and the countries and regions included in the GBD 2019 were divided into five SDI levels: high, middle-high, middle, low-middle, and low (16). The SDI of each country or territory was determined using the GHDx (http://ghdx.healthdata.org/record/ihme-data/gbd-2019-socio-demographic-index-sdi-1950-2019). In addition, the world is geographically divided into 21 regions based on GBD 2019 (Table 1).

**Table 1:** The age-standardized rates for YLDs, YLLs, and DALYs of Down syndrome in 2019 and their temporal trends from 2010 to 2019.

**Characteristics**	**YLDs**			**YLLs**			**DALYs**		
	**ASR per 100,000**	**Change between 2010 and 2019 No. (95% UI)**	**EAPC**	**ASR per 100,000**	**Change between 2010 and 2019 No. (95% UI)**	**EAPC**	**ASR per 100,000**	**Change between 2010 and 2019 No. (95% UI)**	**EAPC**
	**No. (95% UI)**		**No. (95% CI)**	**No. (95% UI)**		**No. (95% CI)**	**No. (95% UI)**		**No. (95% CI)**
Overall	1.87 (1.2 to 2.79)	0.08 (0.03 to 0.13)	1.07 (0.45 to 1.69)	24.15 (18.09 to 38.87)	−0.06 (−0.22 to 0.12)	−0.71 (−0.85 to −0.57)	26.02 (19.83 to 40.75)	−0.05 (−0.21 to 0.11)	−0.59 (−0.69 to −0.49)
**Sex**									
Male	2.03 (1.3 to 3.02)	0.07 (0.02 to 0.13)	1.06 (0.45 to 1.68)	23.71 (18.41 to 43.11)	−0.03 (−0.23 to 0.15)	−0.42 (−0.55 to −0.28)	25.74 (20.22 to 45.11)	−0.03 (−0.21 to 0.15)	−0.31 (−0.41 to −0.21)
Female	1.71 (1.09 to 2.56)	0.08 (0.03 to 0.13)	1.07 (0.44 to 1.7)	24.62 (16.4 to 42.53)	−0.09 (−0.26 to 0.12)	−1 (-1.17 to −0.83)	26.33 (18.11 to 44.13)	−0.08 (−0.25 to 0.11)	−0.88 (−1 to −0.76)
**Socio-demographic index**									
Low	1.17 (0.73 to 1.79)	0.14 (0.08 to 0.2)	1.74 (1.16 to 2.32)	34.26 (17.65 to 77.48)	0 (−0.22 to 0.34)	0.08 (−0.22 to 0.37)	35.43 (18.96 to 78.49)	0 (-0.22 to 0.33)	0.13 (−0.15 to 0.41)
Low-middle	1.28 (0.81 to 1.94)	0.15 (0.1 to 0.2)	1.81 (1.17 to 2.46)	22.51 (16.3 to 33.58)	−0.07 (−0.3 to 0.19)	−0.9 (−1.1 to −0.7)	23.79 (17.59 to 34.96)	−0.06 (-0.29 to 0.18)	−0.77 (−0.93 to −0.61)
Middle	1.71 (1.1 to 2.59)	0.12 (0.07 to 0.18)	1.6 (0.79 to 2.41)	19.24 (15.93 to 23.26)	−0.15 (−0.33 to 0.02)	−1.94 (−2.24 to −1.63)	20.96 (17.75 to 24.94)	−0.13 (−0.31 to 0.03)	−1.69 (−1.95 to −1.43)
Middle-high	2.25 (1.45 to 3.39)	0.04 (-0.02 to 0.11)	0.79 (0.35 to 1.24)	20.09 (17.07 to 24.14)	−0.16 (−0.28 to −0.03)	−2.04 (−2.62 to −1.46)	22.34 (19.33 to 26.28)	−0.14 (−0.26 to −0.02)	−1.78 (−2.3 to −1.25)
High	4.28 (2.77 to 6.24)	0.08 (0 to 0.15)	1.01 (0.4 to 1.63)	16.6 (13.27 to 18.95)	0.03 (−0.05 to 0.11)	0.51 (0.36 to 0.67)	20.88 (17.27 to 23.75)	0.04 (-0.03 to 0.11)	0.61 (0.4 to 0.83)
**Region**									
Asia Pacific to high income	5.15 (3.23 to 7.58)	−0.01 (−0.08 to 0.06)	0.53 (−0.36 to 1.42)	6.59 (4.92 to 7.91)	−0.02 (−0.16 to 0.11)	−0.09 (−0.33 to 0.15)	11.75 (9.22 to 14.52)	−0.01 (−0.1 to 0.07)	0.18 (−0.3 to 0.67)
Central Asia	1.66 (1.03 to 2.52)	0.06 (−0.03 to 0.15)	1.02 (0.09 to 1.96)	7.72 (5.49 to 10.2)	−0.12 (−0.34 to 0.12)	−1.48 (−1.66 to −1.3)	9.39 (7.01 to 11.87)	−0.09 (−0.29 to 0.11)	−1.07 (−1.26 to −0.88)
East Asia	1.24 (0.77 to 1.89)	0.07 (−0.01 to 0.15)	0.85 (0.27 to 1.44)	14.82 (11.34 to 19.56)	−0.35 (−0.51 to −0.18)	−4.95 (−6.06 to −3.82)	16.05 (12.53 to 20.77)	−0.33 (−0.48 to −0.17)	−4.59 (−5.66 to −3.52)
South Asia	1 (0.62 to 1.5)	0.17 (0.12 to 0.23)	2.1 (1.41 to 2.79)	13.47 (8.08 to 19.93)	−0.07 (−0.32 to 0.22)	−0.85 (−1.06 to −0.65)	14.47 (8.99 to 20.91)	−0.06 (−0.3 to 0.22)	−0.67 (−0.83 to −0.51)
Southeast Asia	1.9 (1.2 to 2.86)	0.2 (0.13 to 0.29)	2.26 (1.46 to 3.07)	13.64 (10.84 to 17.06)	−0.02 (−0.28 to 0.26)	−0.18 (−0.39 to 0.04)	15.54 (12.67 to 18.94)	0 (−0.25 to 0.26)	0.09 (−0.04 to 0.22)
Australasia	4.41 (2.8 to 6.54)	0.06 (−0.12 to 0.25)	0.79 (0.49 to 1.09)	20.35 (14.41 to 25.49)	−0.05 (−0.23 to 0.15)	−0.61 (−0.92 to −0.3)	24.76 (18.38 to 29.78)	−0.03 (−0.18 to 0.13)	−0.37 (−0.62 to −0.13)
Caribbean	3.15 (2.02 to 4.76)	0.08 (−0.01 to 0.17)	1.12 (0.57 to 1.67)	46.39 (23.32 to 79.53)	0 (−0.29 to 0.35)	−0.14 (−0.4 to 0.12)	49.55 (26.07 to 82.6)	0.01 (−0.26 to 0.34)	−0.07 (−0.29 to 0.16)
Central Europe	2.09 (1.35 to 3.08)	0.28 (0.18 to 0.42)	2.92 (2.62 to 3.22)	10.45 (8.07 to 13.51)	−0.08 (−0.26 to 0.13)	−0.78 (−0.99 to −0.57)	12.55 (10.05 to 15.5)	−0.03 (−0.2 to 0.15)	−0.25 (−0.45 to −0.05)
Eastern Europe	2.29 (1.47 to 3.48)	−0.05 (−0.16 to 0.01)	0.02 (−0.79 to 0.83)	12.53 (9.21 to 17.03)	0.01 (−0.15 to 0.23)	−0.01 (−1.07 to 1.07)	14.82 (11.21 to 19.13)	0 (−0.13 to 0.18)	0 (−0.89 to 0.9)
Western Europe	5.78 (3.83 to 8.42)	0.11 (0.03 to 0.2)	1.28 (0.8 to 1.77)	19.16 (14.43 to 22.25)	0.02 (−0.08 to 0.12)	0.39 (0.17 to 0.61)	24.94 (19.99 to 28.64)	0.04 (−0.04 to 0.12)	0.59 (0.44 to 0.74)
Andean Latin America	1.59 (1 to 2.4)	0.1 (0 to 0.19)	1.2 (0.67 to 1.73)	28.95 (19.22 to 39.77)	−0.22 (−0.45 to 0.11)	−2.8 (−3.53 to −2.06)	30.55 (21.02 to 41.86)	−0.21 (−0.44 to 0.11)	−2.62 (−3.35 to −1.89)
Central Latin America	2.25 (1.44 to 3.38)	0.04 (−0.03 to 0.13)	0.96 (0.2 to 1.71)	25.92 (18.01 to 34.21)	−0.02 (−0.25 to 0.26)	−0.31 (−0.82 to 0.19)	28.17 (20.16 to 36.52)	−0.01 (−0.23 to 0.24)	−0.22 (−0.65 to 0.22)
Southern Latin America	5.59 (3.54 to 8.53)	−0.09 (−0.26 to 0.1)	−0.49 (−1.19 to 0.22)	40.69 (29.39 to 51.5)	−0.08 (−0.27 to 0.15)	−1.05 (−1.24 to −0.86)	46.29 (34.27 to 57.67)	−0.08 (−0.24 to 0.12)	−0.98 (−1.14 to −0.82)
Tropical Latin America	2.63 (1.69 to 4.05)	0.21 (0.13 to 0.31)	2.31 (1.61 to 3.03)	57.51 (43.75 to 82.75)	−0.06 (−0.24 to 0.18)	−0.9(−1.32 to −0.48)	60.14 (46.55 to 85.79)	−0.05 (−0.23 to 0.19)	−0.78 (−1.2 to −0.36)
North Africa and Middle East	2.93 (1.82 to 4.45)	0.11 (0.05 to 0.19)	1.56 (0.76 to 2.36)	40.57 (30.98 to 56.63)	−0.12 (−0.31 to 0.11)	−1.55 (−1.71 to −1.38)	43.5 (34 to 58.89)	−0.11 (−0.3 to 0.11)	−1.36 (−1.54 to −1.19)
North America to high income	3.44 (2.23 to 4.96)	0.17 (0.04 to 0.3)	1.57 (0.65 to 2.5)	15.68 (12.8 to 17.64)	0.04 (−0.04 to 0.11)	0.62 (0.34 to 0.9)	19.12 (15.97 to 21.43)	0.06 (−0.01 to 0.12)	0.78 (0.47 to 1.09)
Oceania	1.29 (0.79 to 1.97)	0.14 (0.04 to 0.24)	1.69 (1.07 to 2.3)	26.66 (11.1 to 68.75)	0 (−0.32 to 0.55)	−0.01 (−0.18 to 0.16)	27.95 (12.46 to 69.94)	0.01 (−0.3 to 0.52)	0.06 (−0.12 to 0.25)
Central Sub-Saharan Africa	1.06 (0.66 to 1.59)	0.09 (−0.01 to 0.2)	1.11 (0.67 to 1.56)	30.15 (16.04 to 60.79)	−0.15 (−0.51 to 0.52)	−1.94 (−2.4 to −1.47)	31.2 (17.04 to 61.73)	−0.15 (−0.5 to 0.51)	−1.85 (−2.29 to −1.41)
Eastern Sub-Saharan Africa	0.9 (0.57 to 1.36)	0.13 (0.07 to 0.2)	1.65 (1.1 to 2.2)	30.89 (16.19 to 63.77)	−0.01 (−0.26 to 0.41)	−0.13 (−0.5 to 0.24)	31.79 (17.16 to 64.79)	0 (−0.25 to 0.4)	−0.09 (−0.43 to 0.26)
Southern Sub-Saharan Africa	1.68 (1.07 to 2.55)	0.1 (0.02 to 0.2)	1.59 (0.6 to 2.58)	32.85 (24.55 to 42.8)	0.18 (−0.09 to 0.52)	1.81 (1.18 to 2.44)	34.53 (26.36 to 44.44)	0.18 (−0.08 to 0.5)	1.79 (1.23 to 2.36)
Western Sub-Saharan Africa	0.98 (0.62 to 1.48)	0.09 (0.05 to 0.14)	1.15 (0.72 to 1.58)	43.03 (16.09 to 114.51)	−0.01 (−0.24 to 0.44)	0.15 (−0.38 to 0.69)	44.01 (17.28 to 115.77)	−0.01 (−0.23 to 0.42)	0.17 (−0.35 to 0.7)

*YLDs, years Lived with Disability; YLLs, years of Life Lost; DALYs, disability-Adjusted Life Years; ASR, age-standardized rate; CI, confidence interval; EAPC, estimated annual percentage change; UI, uncertainty interval*.

### Statistical Analysis

The ASRs and estimated annual percentage changes (EAPCs) of YLDs, YLLs, and DALYs were used to evaluate their trends from 2010 to 2019, and the DS burden. EAPC was introduced as a concept to describe ASR trends within a specified time interval. The natural logarithm of ASR is assumed to vary linearly with time; in the formula Y = α + βX + ε, where Y is ln(ASR), X is the calendar year, and ε is an error term. From this formula, β determines whether there is a positive or negative trend in ASR. EAPC is calculated as 100 × (exp(β) – 1). From the linear model, 95% confidence intervals (CIs) are obtained. A positive EAPC and lower bound of the CI indicated that the ASR is exhibiting an upward trend, while negative EAPC and upper bound indicate a decreasing trend; otherwise ASR is considered stable. However, ASR is still considered stable regardless of EAPC when the change between 2010 and 2019 is not significant. We also evaluated the relationship between ASR and SDI using locally weighted regression (Loess) and Pearson's correlation coefficient. In Loess, when estimating the value of a response variable, a linear regression is performed based on the points near the response variable. The regression adopts the weighted least square method (the closer the value is to the estimation point, the greater the weight is). Finally, the obtained local regression model is used to estimate the value of the response variable. The distribution of weight is Gaussian distribution. Statistical analyses were performed using R software (version 3.4.3), and the criterion for statistical significance was *p* < 0.05.

## Results

### Global DS Burden

**Figure 1:**
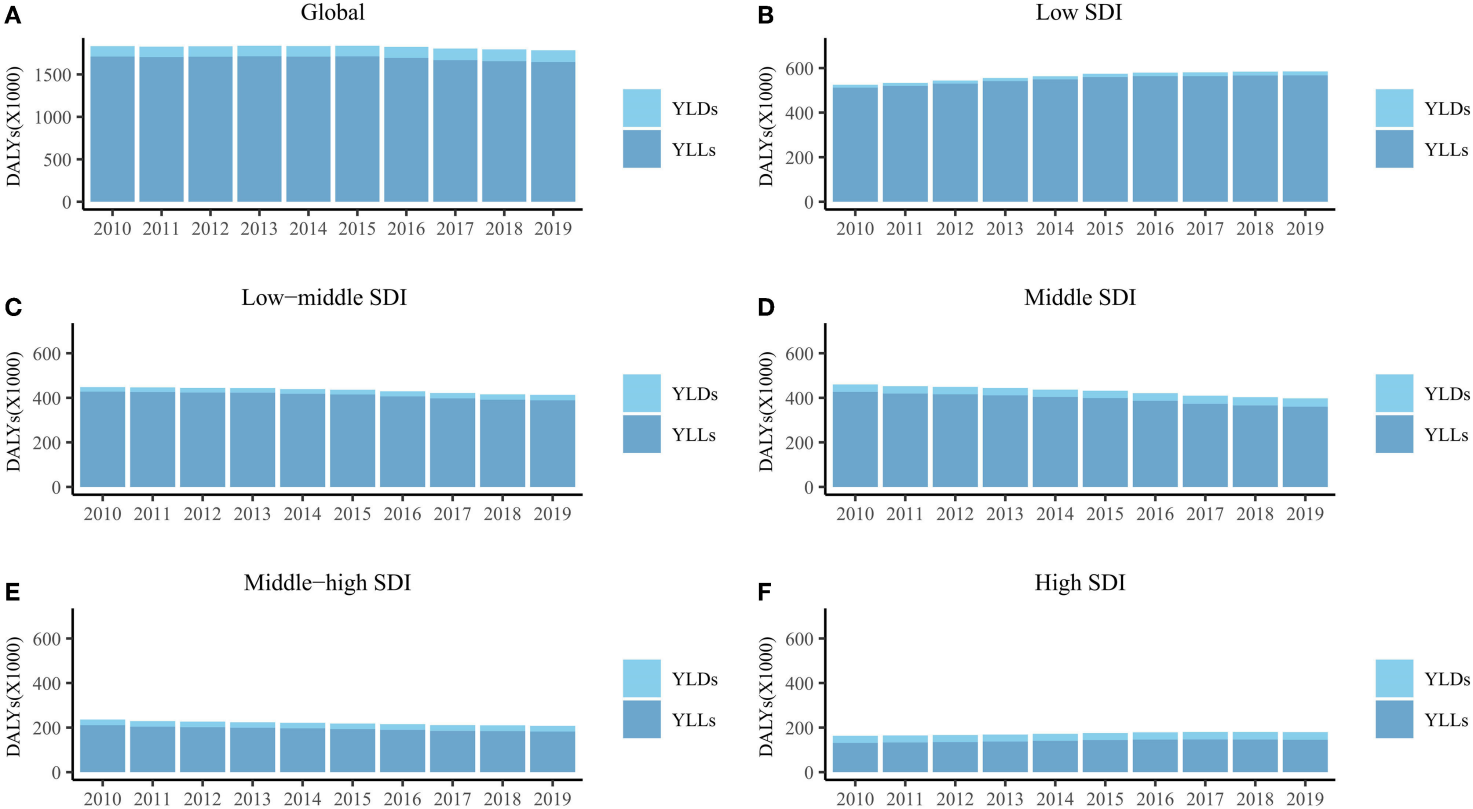
The DALYs, YLDs, and YLLs of Down syndrome for global **(A)**, low SDI region **(B)**, low-middle SDI region **(C)**, middle SDI region **(D)**, middle-high SDI region **(E)** and high SDI region **(F)** from 2010 to 2019. DALYs, disability-adjusted life years; YLDs, years lived with disability; YLLs, years of life lost; SDI, socio-demographic index.

The total global DALYs due to DS decreased by 1.78 million (2.68%) from 2010 to 2019 (95 % UI = 1.37 to 2.75 million) (Figure 1A). The ASR of DALYs was stable (EAPC = −0.59, 95% CI = −0.69 to −0.49), mostly due to the stability of ASR for YLLs (EAPC = −0.71, 95% CI = −0.85 to −0.57), whereas the ASR of YLDs exhibited an increasing trend (EAPC = 1.07, 95% CI = 0.45 to 1.69) (Table 1).

In the five SDI regions, DALYs increased in the low- and high-SDI regions from 2010 to 2019, but decreased in the other three SDI regions (Figures 1B–F). DALYs were mostly attributable to YLLs in all SDI regions (Figures 1B–F). The ASR of YLLs and DALYs decreased only from 2010 to 2019 (YLLs, EAPC = −2.04, 95% CI = −2.62 to −1.46; DALYs, EAPC = 0.61, 95% CI = 0.40 to 0.83) in the middle-high-SDI regions (Table 1). The ASR of YLDs in the middle-high-SDI regions was stable from 2010 to 2019, and increased in the remaining four SDI regions (Table 1).

**Figure 2:**
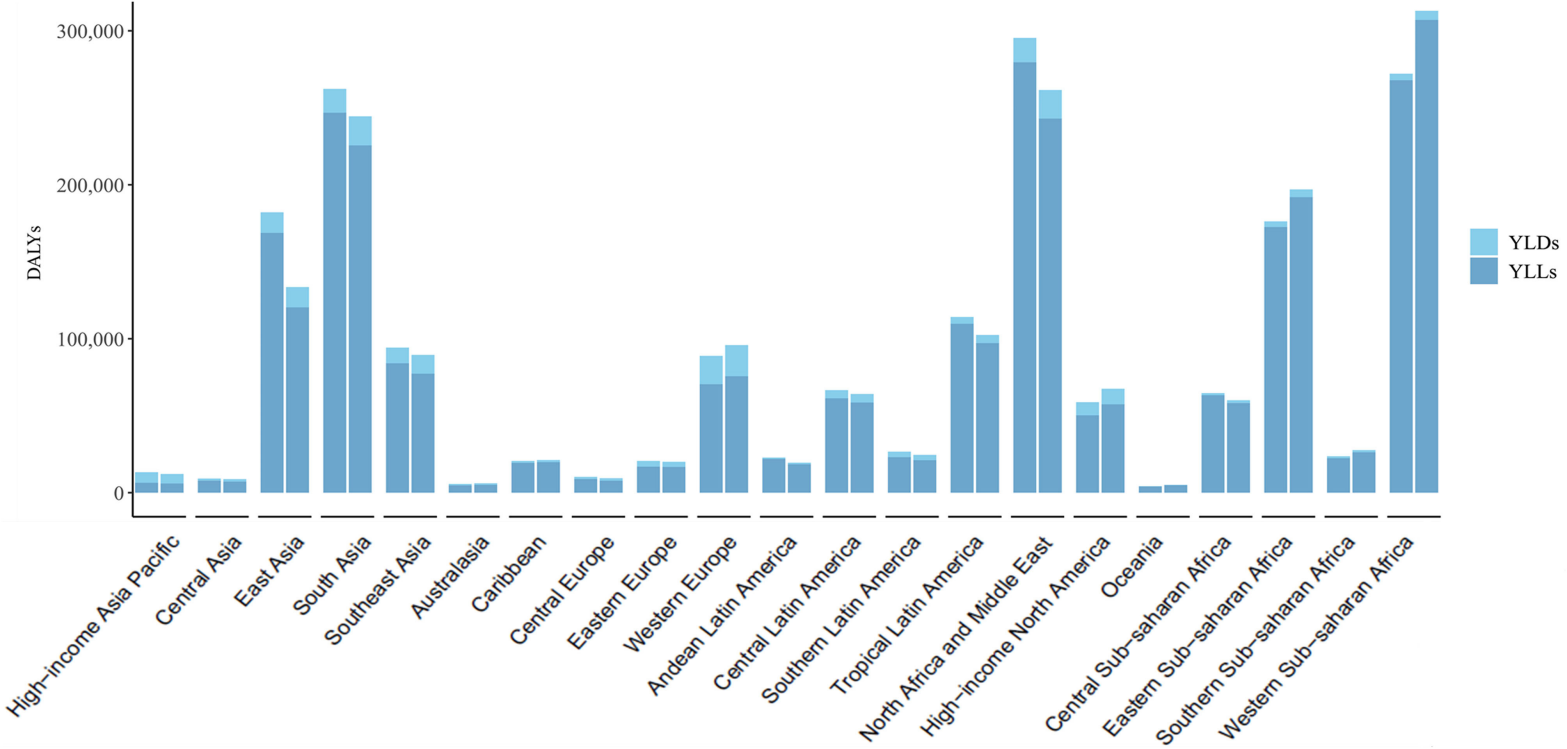
The DALYs, YLDs, and YLLs of Down syndrome at a regional level. The left column in each group is the data in 2010 and the right column in 2019. DALYs, disability-adjusted life years; YLDs, years lived with disability; YLLs, years of life lost.

The DALYs and ASRs differed among the 21 regions (Figure 2; Table 1). In addition to Asia Pacific–high income, DALYs in other regions were mostly attributable to YLLs (Figure 2). DALYs were lower in 2019 than in 2010 in most of 21 regions (Figure 2). The ASR of DALYs in 2019 was the highest in Tropical Latin America (60.14, 95% UI = 46.55 to 85.79) and lowest in Central Asia (9.39, 95% UI = 7.01 to 11.87) (Table 1). The ASR of DALYs or YLLs in most (20 of 21) regions exhibited a stable trend from 2010 to 2019. This trend was decreasing only in East Asia. However, The ASR of YLDs is increasing in 12 regions including South Asia, Southeast Asia, Central Europe, Western Europe, Andean Latin America, Tropical Latin America, North Africa and Middle East, North America–high income, Oceania, Eastern Sub-Saharan Africa, Southern Sub-Saharan Africa, and Western Sub-Saharan Africa. The remaining 9 regions exhibited stable trends in their ASRs of YLDs (Table 1).

The DS burden varied between countries and territories (Supplementary Table S1). The trend of ASRs of DALYs was stable in most countries (192 of 204), decreased in 4, and increased in the remaining 8 (Supplementary Table S1).

### DS Burden Distribution by Age

**Figure 3:**
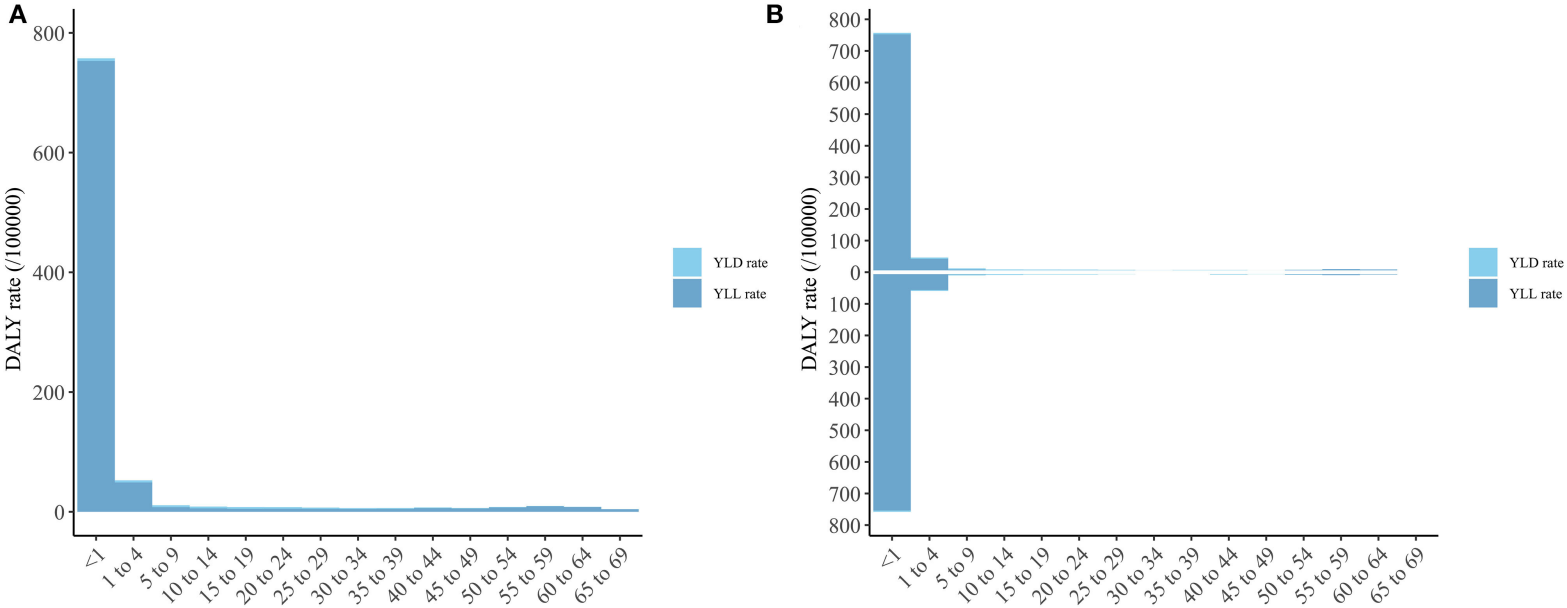
The DALY, YLD, and YLL rate of Down syndrome of different age group. **(A)** Both sexes; **(B)** Male and Female (The column above is for male and the column below is for female). DALY, disability-adjusted life year; YLD, year lived with disability; YLL, year of life lost.

As shown in Figure 3, the DS DALY rate was mostly attributable to the YLLs of children younger than 1 year, followed by those aged 1–4 years, and there was no significant difference in DS burden between males and females.

### Relationship Between ASR and SDI

**Figure 4:**
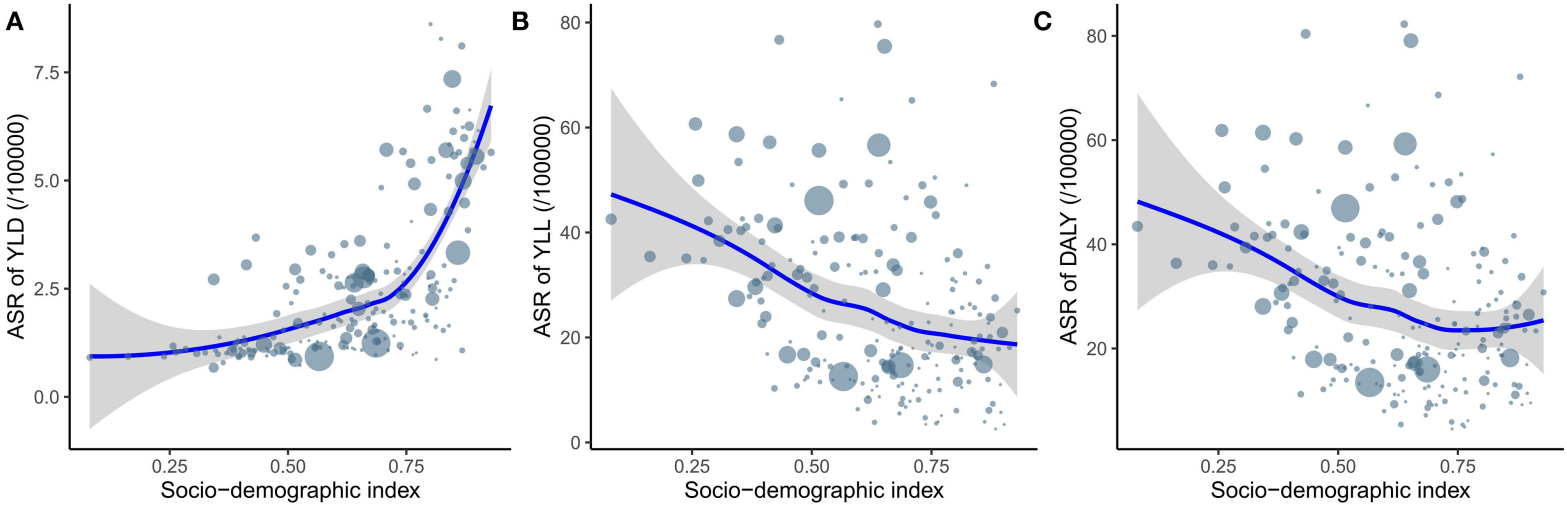
The relationship between ASR of YLD **(A)**, YLL **(B)** and DALY **(C)** and SDI. ASR, age-standardized rate; YLD, year lived with disability; YLL, year of life lost; DALY, disability-adjusted life year; SDI, socio-demographic index.

Figure 4 shows the relationship between ASR and SDI for the 204 countries and territories. There was a significant positive association (ρ = 0.62, *p* < 0.001) between the ASR of YLDs and SDI, a significant negative association (ρ = −0.37, *p* < 0.001) between the ASR of YLLs and SDI, and a significant negative association (ρ = −0.3, *p* < 0.001) between the ASR of DALYs and SDI. From Figure 4 it can been seen that the ASR of YLDs increased more when SDI exceeded 0.75, which led to the stability of the ASR of DALYs after this point.

## Discussion

DS is the most-common chromosomal disease in children, with a reported prevalence in live births of 0.07–0.2%. DS exerts many adverse effects on affected patients (17, 18). DS is the most frequent cause of intellectual disability due to chromosomal disorders and, unlike other neurodevelopmental disorders, it lasts throughout life. Thus, DS is a severe social burden. However, the disease burden of DS in different regions or countries is different and unclear. This study examined the global disease burden of DS using three types of distribution (time, geographical, and population), and analyzed the relationship between the disease burden of DS and SDI based on the DS data in the GBD database.

This study found that DALYs decreased worldwide by 2.68% from 2010 to 2019. After age standardization, the DALY rate decreased by an average of 0.71% annually over this period. However, there was notable regional imbalance. Most of DALYs or their ASRs were attributable to areas with relatively low SDI. This may be due to the lack of good medical conditions and policies in low-SDI regions and countries. DALYs are calculated as the sum of YLDs and YLLs, which are attributable to death and disability, respectively. The main method used to reduce the disease burden of DS is prenatal neonatal disease screening and DS-related disease screening, prevention and treatment, which require a series of medical policies, technology, and condition support that can be very difficult to provide in low-SDI regions and countries. Our analysis of the relationship between SDI and DALY rates indicated that DALY rates decrease as SDI increases. Take China as an example. Although its fertility rate is declining (19), the education level and per capita income of the population have increased significantly (20, 21). Accordingly, in the GBD database, its SDI increased from 0.621 in 2010 to 0.686 in 2019. In this study, its DALY rate decreased by 34% with an EAPC of −4.67 in 10 years. It was particularly interesting that the YLD and YLL rates exhibited the opposite trend. That is, the YLL rate decreased as SDI increased and YLD rates increased alongside SDI. For example, the high-SDI regions had the lowest YLL rates but the highest YLD rates among the five SDI regions. This phenomenon can also be observed at the geographical and national levels. This may be due to high levels of medical conditions reducing the mortality of DS and thus reducing YLL rates, but the risk of disability increases due to the increased survival duration for children with DS, which increases YLD rates. According to the current results, the main method used to reduce DALYs in areas with low SDI should still be reducing deaths from DS. Prenatal neonatal disease screening is a more-suitable and realistic method since patients with DS have many complications, and it is more difficult to establish a complete set of technical methods for these complications than using neonatal screening to prevent DS death in areas with low SDI.

In addition, The DALY rates in this study were mostly attributable to the YLL rates of people younger than 1 year, and so more attention to screening DS-related diseases or death risk in this age range may be more effective in reducing YLL rates. For example, DS can present alongside various congenital malformations including congenital heart disease, was well as abnormalities of the digestive system, musculoskeletal, respiratory, and urinary systems (22). Congenital heart disease is the most common of these conditions, being present in 40~60% of DS cases, and is the most-common cause of death in patients with DS (23). Due to the characteristics of heart defects and respiratory hypoplasia, children with DS are prone to early pulmonary hypertension formation, leading to pulmonary vascular disease and heart failure, which seriously affect the prognoses of patients with DS. Early diagnosis and intervention of congenital heart disease is therefore very important for prolonging the survival of children with DS (24); this may reduce the YLL rate, in turn decreasing the disease burden of DS. Notably, although high-SDI regions had the lowest DS DALY rates among the five SDI regions, they were the only regions with increasing trends in YLL and YLD rates from 2010 to 2019. More research is required to explain the cause of this phenomenon, which is currently unknown. Of course, SDI is only one of the factors affecting the disease burden of DS. For example, social values are another factor affecting its disease burden. For the population with DS diagnosed via neonatal disease screening, pregnancy termination is the most-effective means to reduce the disease burden of DS, but pregnancy termination is affected by variations in social values (e.g., law, morality, religion, and ethics) among countries or regions. This can also partly be explained by how regions and countries with similar SDIs have relatively large differences in the disease burden of DS.

This study was subject to some notable limitations. First, only the global disease burden of DS was analyzed, with the different risk factors for DS in different countries and region**s** not being addressed. Because the risk factors affecting YLD, YLL and DALY rate of DS cannot be extracted in the GHDx. Second, EAPC estimation comes from point estimate with CI rather than the interval estimate with UI. EAPC with CI calculated based on GBD data is not appropriate because the estimates of GBD data are from interval estimation with UI. In addition, in order to make the DS burden trend obtained by EAPC more conservative, we use DS burden change with UI to control it.

This study performed the most-comprehensive assessment of the disease burden of DS from 2010 to 2019. Although the global DS DALY rate was found to be stable, there were significant differences for DS burden among regions or countries. Future analyses of how to control the burden of DS should pay attention to DS-related deaths in children younger than 1 year and the DS burden in low-SDI regions and countries. The information yielded by this study should help in understanding the global disease burden of DS and in the establishment of more-effective and targeted strategies or measures that could further reduce the DS burden.

## Data Availability Statement

The raw data supporting the conclusions of this article will be made available by the authors, without undue reservation.

## Ethics Statement

This study was performed in accordance with the Declaration of Helsinki and was approved by the Institutional Review Board of Xi'an Children's Hospital.

## Author Contributions

QB and ZP: study design and data extraction. ZP: statistical analysis. QB, RQ, HCheng, AW, HChen, and ZP: manuscript draft. All authors approved the final version.

## Funding

This study was supported by the Natural Science Basic Research Program of Shaanxi (No. 2020JQ-929) and the Research Fund of Xi'an Children's Hospital (No. 2021G03).

## Conflict of Interest

The authors declare that the research was conducted in the absence of any commercial or financial relationships that could be construed as a potential conflict of interest.

## Publisher's Note

All claims expressed in this article are solely those of the authors and do not necessarily represent those of their affiliated organizations, or those of the publisher, the editors and the reviewers. Any product that may be evaluated in this article, or claim that may be made by its manufacturer, is not guaranteed or endorsed by the publisher.
