# Supplementary material for: Analysis of the Global Disease Burden of Down Syndrome Using YLDs, YLLs, and DALYs Based on the Global Burden of Disease 2019 Data
Source: Front Pediatr. 2022 Apr 29;10:882722. doi: 10.3389/fped.2022.882722 (PMC9099075; doi:10.3389/fped.2022.882722)
Supplement: Supplementary file 1 [file Table_1.docx]

**Table S1** The age-standardized rates for YLDs, YLLs and DALYs of Down syndrome in 2019 and their temporal trends from 2010 to 2019 at country and territory level

| **Country and territory** | **YLDs** | | |  | **YLLs** | | |  | **DALYs** | | |
| --- | --- | --- | --- | --- | --- | --- | --- | --- | --- | --- | --- |
|  | **ASR per 100,000** | **Change between 2010 and 2019** | **EAPC** |  | **ASR per 100,000** | **Change between 2010 and 2019** | **EAPC** |  | **ASR per 100,000** | **Change between 2010 and 2019** | **EAPC** |
|  | **No.(95% UI)** |  | **No.(95% CI)** |  | **No.(95% UI)** |  | **No.(95% CI)** |  | **No.(95% UI)** |  | **No.(95% CI)** |
| Afghanistan | 2.71(1.51–4.43) | 0.2(0.03–0.45) | 2.47(1.52–3.42) |  | 58.71(26.65–114.82) | -0.28(-0.58–0.36) | -3.78(-4.03–-3.53) |  | 61.42(29.33–117.45) | -0.27(-0.57–0.34) | -3.57(-3.78–-3.36) |
| Albania | 1.62(1.03–2.43) | 0.08(-0.05–0.2) | 0.97(0.69–1.26) |  | 10.73(6.43–17.96) | -0.13(-0.41–0.29) | -1.72(-2.05–-1.39) |  | 12.36(8.04–19.4) | -0.11(-0.36–0.26) | -1.41(-1.67–-1.14) |
| Algeria | 3.61(2.22–5.54) | 0.15(0.01–0.32) | 1.86(1.3–2.42) |  | 75.47(46–125.99) | -0.11(-0.4–0.41) | -1.72(-2.12–-1.32) |  | 79.08(49.14–128.46) | -0.1(-0.38–0.4) | -1.58(-1.95–-1.2) |
| American Samoa | 1.06(0.67–1.61) | 0.06(-0.06–0.19) | 0.74(0.31–1.17) |  | 9.9(5.9–15.61) | -0.28(-0.55–0.15) | -4.92(-6.46–-3.36) |  | 10.97(6.9–16.83) | -0.25(-0.52–0.14) | -4.49(-5.92–-3.04) |
| Andorra | 5.89(3.81–8.67) | 0.12(-0.01–0.26) | 1.51(1–2.02) |  | 11.02(6.82–17.87) | 0.02(-0.26–0.35) | 0.24(-0.45–0.94) |  | 16.91(12–24.37) | 0.05(-0.15–0.28) | 0.66(0.17–1.15) |
| Angola | 1.02(0.64–1.53) | 0.09(-0.04–0.23) | 1.13(0.67–1.59) |  | 31.99(15.39–65.16) | -0.18(-0.57–0.68) | -2.35(-2.55–-2.16) |  | 33.01(16.53–66.43) | -0.17(-0.56–0.66) | -2.26(-2.45–-2.07) |
| Antigua and Barbuda | 2.65(1.69–3.86) | 0.13(-0.01–0.29) | 1.58(0.91–2.24) |  | 16.02(11.17–22.09) | 0.01(-0.23–0.32) | 0.11(-0.4–0.62) |  | 18.67(13.83–24.76) | 0.03(-0.18–0.3) | 0.3(-0.08–0.69) |
| Argentina | 5.71(3.59–8.82) | -0.04(-0.28–0.2) | 0.5(-0.65–1.66) |  | 39.08(28.84–50.47) | -0.04(-0.26–0.26) | -0.41(-0.82–0.01) |  | 44.79(34.07–56.65) | -0.04(-0.23–0.22) | -0.28(-0.64–0.07) |
| Armenia | 1.52(0.95–2.26) | 0.13(0–0.37) | 1.72(1.08–2.37) |  | 11.22(8.56–15.59) | -0.01(-0.28–0.37) | 0.1(-0.47–0.67) |  | 12.74(9.94–17.08) | 0(-0.24–0.35) | 0.28(-0.28–0.85) |
| Australia | 4.28(2.7–6.38) | 0.06(-0.15–0.3) | 0.83(0.55–1.1) |  | 19.34(13.85–24.1) | -0.02(-0.2–0.19) | -0.23(-0.64–0.19) |  | 23.62(17.71–28.54) | 0(-0.15–0.17) | -0.04(-0.37–0.28) |
| Austria | 6.14(3.93–9.17) | 0.06(-0.07–0.22) | 0.8(0.62–0.98) |  | 30.65(21.68–36.21) | -0.08(-0.27–0.11) | -0.9(-1.11–-0.69) |  | 36.79(27.18–43.34) | -0.06(-0.22–0.1) | -0.64(-0.84–-0.44) |
| Azerbaijan | 1.66(1.02–2.54) | 0.12(0–0.28) | 1.54(0.84–2.24) |  | 5.52(2.95–8.94) | -0.21(-0.5–0.27) | -2.7(-3.02–-2.38) |  | 7.18(4.5–10.66) | -0.15(-0.41–0.23) | -1.86(-2.24–-1.47) |
| Bahamas | 2.74(1.75–4.1) | 0.11(-0.03–0.29) | 1.38(0.81–1.95) |  | 24.7(17.97–32.99) | -0.01(-0.23–0.27) | -0.3(-0.61–0.02) |  | 27.44(20.83–35.86) | 0(-0.2–0.25) | -0.14(-0.41–0.13) |
| Bahrain | 2.34(1.46–3.44) | 0.19(0.03–0.38) | 2.2(1.75–2.65) |  | 18.54(12.61–26.69) | 0.49(0.03–1.16) | 4.39(2.27–6.55) |  | 20.87(14.78–29.28) | 0.45(0.05–1) | 4.12(2.28–6) |
| Bangladesh | 1.12(0.68–1.72) | 0.15(0.02–0.28) | 1.71(1.16–2.26) |  | 16.77(8.96–28.17) | -0.14(-0.49–0.37) | -1.61(-1.79–-1.42) |  | 17.89(9.93–29.22) | -0.13(-0.47–0.36) | -1.43(-1.61–-1.25) |
| Barbados | 2.52(1.64–3.79) | 0.09(-0.05–0.23) | 1.06(0.73–1.4) |  | 26.47(17.85–36.3) | -0.04(-0.28–0.26) | -0.51(-0.66–-0.36) |  | 28.99(20.27–39.02) | -0.03(-0.25–0.25) | -0.38(-0.53–-0.23) |
| Belarus | 2.35(1.46–3.61) | 0.06(-0.08–0.23) | 0.87(0.18–1.56) |  | 9.76(5.89–15.84) | 0.07(-0.23–0.46) | 1.09(0.58–1.61) |  | 12.11(8.22–18.25) | 0.07(-0.17–0.39) | 1.05(0.53–1.56) |
| Belgium | 5.58(3.56–8.17) | 0.12(-0.03–0.28) | 1.44(1.01–1.88) |  | 20.21(14.6–24.27) | -0.04(-0.21–0.14) | -0.43(-0.65–-0.21) |  | 25.79(20.03–30.81) | -0.01(-0.15–0.14) | -0.05(-0.22–0.11) |
| Belize | 2.76(1.77–4.1) | 0.1(-0.05–0.27) | 1.27(0.84–1.71) |  | 32.12(22.41–41.52) | -0.03(-0.24–0.24) | -0.41(-0.65–-0.16) |  | 34.88(25.07–44.05) | -0.02(-0.22–0.23) | -0.28(-0.52–-0.04) |
| Benin | 0.98(0.62–1.48) | 0.05(-0.05–0.17) | 0.61(0.39–0.83) |  | 40.36(15.86–95.24) | -0.04(-0.41–0.72) | -0.34(-1.09–0.42) |  | 41.34(16.82–96.2) | -0.03(-0.4–0.7) | -0.32(-1.05–0.42) |
| Bermuda | 2.05(1.29–3.05) | 0.06(-0.09–0.2) | 0.72(0.45–0.99) |  | 16.1(11.59–21.94) | 0.09(-0.17–0.41) | 1.07(0.82–1.32) |  | 18.15(13.74–24.1) | 0.08(-0.15–0.36) | 1.03(0.82–1.24) |
| Bhutan | 1.12(0.69–1.68) | 0.05(-0.06–0.16) | 0.61(0.35–0.88) |  | 19.17(9.97–33.9) | -0.11(-0.48–0.48) | -1.26(-1.81–-0.72) |  | 20.29(10.94–34.97) | -0.11(-0.46–0.46) | -1.17(-1.7–-0.64) |
| Bolivia (Plurinational State of) | 1.72(1.07–2.59) | 0.11(-0.02–0.24) | 1.35(0.8–1.9) |  | 49.22(29.53–76.95) | -0.15(-0.49–0.41) | -1.77(-1.92–-1.63) |  | 50.93(31.26–78.4) | -0.15(-0.48–0.39) | -1.68(-1.83–-1.54) |
| Bosnia and Herzegovina | 1.87(1.15–2.72) | 0.12(-0.05–0.36) | 1.29(1.23–1.36) |  | 10.84(7.66–15.29) | -0.14(-0.41–0.27) | -1.81(-3.02–-0.58) |  | 12.71(9.49–17.09) | -0.11(-0.36–0.25) | -1.41(-2.47–-0.34) |
| Botswana | 1.8(1.12–2.74) | 0.07(-0.04–0.22) | 1.13(0.41–1.85) |  | 32.54(19.86–52.16) | 0.11(-0.27–0.68) | 1.1(0.86–1.33) |  | 34.34(21.71–54.16) | 0.11(-0.25–0.65) | 1.1(0.87–1.33) |
| Brazil | 2.64(1.7–4.06) | 0.21(0.13–0.31) | 2.35(1.64–3.08) |  | 56.64(43.18–82.66) | -0.06(-0.25–0.18) | -0.98(-1.42–-0.53) |  | 59.28(45.74–85.29) | -0.05(-0.24–0.18) | -0.85(-1.3–-0.4) |
| Brunei Darussalam | 8.28(5.16–12.05) | 0.11(-0.04–0.31) | 1.46(0.92–2) |  | 48.99(34.15–68.3) | 0.08(-0.26–0.57) | 0.86(0.53–1.2) |  | 57.27(42.05–77.04) | 0.08(-0.23–0.5) | 0.95(0.68–1.21) |
| Bulgaria | 1.98(1.24–2.99) | 0.09(-0.07–0.27) | 1.13(0.86–1.4) |  | 12.13(8.55–17.13) | -0.08(-0.36–0.3) | -0.47(-1.98–1.05) |  | 14.11(10.41–18.97) | -0.05(-0.31–0.27) | -0.26(-1.6–1.09) |
| Burkina Faso | 1.17(0.75–1.76) | 0.07(-0.04–0.2) | 0.79(0.77–0.81) |  | 60.67(16.29–197.15) | 0.26(-0.24–1.26) | 2.99(2.3–3.69) |  | 61.85(17.44–197.93) | 0.25(-0.24–1.23) | 2.95(2.27–3.63) |
| Burundi | 1.11(0.69–1.7) | 0.04(-0.06–0.16) | 0.54(0.17–0.91) |  | 42.22(16.47–125.05) | -0.06(-0.42–0.71) | -1.19(-2.3–-0.06) |  | 43.33(17.46–125.97) | -0.05(-0.42–0.68) | -1.14(-2.23–-0.05) |
| Cabo Verde | 0.73(0.46–1.12) | -0.01(-0.12–0.1) | -0.09(-0.32–0.14) |  | 25.06(11.48–42.73) | -0.06(-0.52–0.66) | -0.37(-1.12–0.37) |  | 25.79(12.31–43.56) | -0.06(-0.51–0.63) | -0.37(-1.08–0.35) |
| Cambodia | 1.38(0.84–2.12) | 0.21(0.08–0.4) | 2.38(1.77–2.99) |  | 10.79(6.26–17.99) | -0.12(-0.49–0.45) | -1.22(-1.76–-0.67) |  | 12.17(7.6–19.13) | -0.09(-0.45–0.42) | -0.87(-1.41–-0.32) |
| Cameroon | 1.05(0.67–1.59) | 0.05(-0.06–0.17) | 0.69(0.42–0.96) |  | 31.43(13.12–77.77) | 0.02(-0.42–0.8) | 0.1(-0.2–0.39) |  | 32.48(14.17–78.68) | 0.02(-0.41–0.76) | 0.11(-0.17–0.4) |
| Canada | 4.48(2.81–6.68) | 0.07(-0.19–0.41) | 0.96(0.58–1.34) |  | 23.83(17.52–27.97) | 0.1(-0.05–0.27) | 1.13(0.55–1.72) |  | 28.31(21.87–33.23) | 0.1(-0.04–0.25) | 1.1(0.63–1.58) |
| Central African Republic | 1.05(0.64–1.6) | 0.09(-0.03–0.23) | 1.09(0.66–1.53) |  | 34.7(11.35–102.04) | -0.09(-0.43–0.57) | -1.04(-1.46–-0.61) |  | 35.75(12.41–103.28) | -0.09(-0.43–0.55) | -0.98(-1.38–-0.58) |
| Chad | 0.93(0.59–1.41) | 0.13(0.02–0.25) | 1.61(1.24–1.98) |  | 35.06(10.24–104.24) | -0.02(-0.41–0.72) | -0.16(-0.35–0.03) |  | 35.99(11.07–105.01) | -0.02(-0.41–0.69) | -0.11(-0.3–0.07) |
| Chile | 5.4(3.42–7.97) | -0.2(-0.34–-0.05) | -3.13(-3.91–-2.35) |  | 43.3(25.83–60.7) | -0.15(-0.42–0.18) | -2.17(-3.24–-1.09) |  | 48.7(30.4–66.11) | -0.15(-0.39–0.14) | -2.28(-3.29–-1.26) |
| China | 1.24(0.78–1.9) | 0.07(-0.01–0.15) | 0.84(0.25–1.43) |  | 14.73(11.35–19.54) | -0.36(-0.51–-0.19) | -5.03(-6.17–-3.87) |  | 15.97(12.42–20.71) | -0.34(-0.49–-0.18) | -4.67(-5.76–-3.56) |
| Colombia | 2.25(1.38–3.45) | 0.01(-0.21–0.25) | 0.47(0.04–0.9) |  | 12.13(7.41–17.39) | 0.33(-0.17–0.96) | 3.91(0.69–7.25) |  | 14.38(9.61–19.78) | 0.27(-0.13–0.77) | 3.32(0.62–6.09) |
| Comoros | 0.95(0.6–1.44) | 0.06(-0.04–0.17) | 0.77(0.44–1.11) |  | 34.75(17.65–68.37) | -0.12(-0.49–0.76) | -1.23(-2.49–0.05) |  | 35.7(18.54–69.39) | -0.12(-0.48–0.73) | -1.18(-2.41–0.06) |
| Congo | 1.15(0.72–1.74) | 0.11(0–0.31) | 1.34(0.88–1.8) |  | 26.9(15.67–49) | -0.02(-0.39–0.62) | -0.22(-0.55–0.12) |  | 28.05(16.85–50.25) | -0.01(-0.38–0.58) | -0.16(-0.46–0.15) |
| Cook Islands | 0.97(0.6–1.49) | 0.1(-0.03–0.25) | 1.13(0.8–1.46) |  | 3.94(1.73–7.68) | -0.29(-0.58–0.06) | -3.8(-4.52–-3.08) |  | 4.91(2.63–8.64) | -0.24(-0.49–0.07) | -3.01(-3.67–-2.34) |
| Costa Rica | 1.94(1.22–2.96) | 0.03(-0.19–0.35) | 0.96(0.29–1.63) |  | 17.17(11.41–24.52) | -0.08(-0.39–0.34) | -1.12(-2.37–0.16) |  | 19.11(13.24–26.41) | -0.07(-0.35–0.31) | -0.91(-2.03–0.22) |
| Côte d'Ivoire | 1.25(0.8–1.9) | 0.05(-0.05–0.16) | 0.64(0.39–0.89) |  | 31.73(13.71–71.13) | 0.04(-0.35–0.83) | 0.69(-0.1–1.48) |  | 32.97(15.01–72.72) | 0.04(-0.34–0.78) | 0.69(-0.06–1.44) |
| Croatia | 1.77(1.11–2.63) | 0.11(-0.04–0.29) | 0.97(0.74–1.21) |  | 31.06(21.89–42.44) | -0.04(-0.31–0.3) | -0.27(-0.83–0.28) |  | 32.83(23.48–44.38) | -0.04(-0.28–0.29) | -0.21(-0.74–0.32) |
| Cuba | 2.17(1.38–3.22) | -0.14(-0.3–0.14) | -1.31(-1.84–-0.78) |  | 16.62(12.1–21.78) | -0.02(-0.23–0.23) | 0.16(-0.39–0.71) |  | 18.79(14.43–24.12) | -0.03(-0.22–0.2) | -0.02(-0.51–0.46) |
| Cyprus | 5.83(3.85–8.56) | 0.08(-0.05–0.23) | 1.11(0.61–1.61) |  | 14.96(11.89–20.19) | 0.02(-0.18–0.3) | 0.28(-0.26–0.82) |  | 20.79(17.04–26.34) | 0.04(-0.12–0.25) | 0.51(0–1.01) |
| Czechia | 1.53(0.94–2.33) | 0.13(-0.02–0.39) | 1.66(1.36–1.96) |  | 11.53(8.22–16.63) | 0.17(-0.13–0.53) | 1.08(-1.02–3.23) |  | 13.06(9.58–18.07) | 0.16(-0.11–0.5) | 1.14(-0.72–3.05) |
| Democratic People's Republic of Korea | 1.16(0.7–1.79) | 0.1(-0.04–0.26) | 1.2(0.58–1.82) |  | 20.04(10.46–35.13) | -0.31(-0.61–0.17) | -4.01(-4.22–-3.81) |  | 21.2(11.36–36.36) | -0.29(-0.58–0.17) | -3.79(-4.02–-3.56) |
| Democratic Republic of the Congo | 1.06(0.66–1.62) | 0.1(-0.03–0.22) | 1.12(0.68–1.56) |  | 29.6(15.08–63.5) | -0.16(-0.56–0.68) | -1.95(-2.62–-1.27) |  | 30.66(16.14–64.63) | -0.15(-0.55–0.65) | -1.86(-2.5–-1.22) |
| Denmark | 5.69(3.71–8.35) | 0.09(-0.05–0.23) | 1.13(0.68–1.59) |  | 17.81(13.83–24.23) | -0.06(-0.23–0.12) | -0.92(-1.64–-0.19) |  | 23.5(18.93–30.01) | -0.03(-0.17–0.11) | -0.46(-0.97–0.04) |
| Djibouti | 1.04(0.66–1.61) | 0.07(-0.04–0.19) | 0.9(0.48–1.31) |  | 49.1(17.49–108.22) | 0.17(-0.3–1.04) | 1.97(1.67–2.27) |  | 50.14(18.5–109.46) | 0.17(-0.29–0.99) | 1.95(1.65–2.24) |
| Dominica | 3.13(1.94–4.68) | 0.17(0.01–0.43) | 2.07(1.44–2.71) |  | 32.21(21.74–46.34) | 0.08(-0.25–0.55) | 0.69(0.47–0.92) |  | 35.34(24.77–49.5) | 0.09(-0.21–0.51) | 0.81(0.64–0.98) |
| Dominican Republic | 3.29(2.05–5.02) | 0.07(-0.06–0.22) | 1.09(0.52–1.66) |  | 39.07(22.13–65.29) | -0.27(-0.56–0.2) | -3.79(-4.54–-3.04) |  | 42.35(25.61–68.43) | -0.25(-0.53–0.18) | -3.49(-4.17–-2.8) |
| Ecuador | 1.71(1.07–2.58) | 0.1(-0.04–0.28) | 1.27(0.74–1.8) |  | 36.05(24.07–51.22) | -0.28(-0.53–0.09) | -3.83(-4.45–-3.2) |  | 37.77(25.46–52.86) | -0.27(-0.51–0.09) | -3.64(-4.26–-3.01) |
| Egypt | 2.9(1.76–4.5) | 0.12(-0.01–0.27) | 1.53(0.68–2.39) |  | 14.44(6.56–29.37) | -0.19(-0.51–0.35) | -2.13(-2.74–-1.51) |  | 17.34(9.24–32.29) | -0.15(-0.45–0.32) | -1.6(-2.21–-0.99) |
| El Salvador | 2.91(1.79–4.45) | 0.1(-0.05–0.28) | 1.29(0.44–2.14) |  | 14.56(6.71–22.78) | -0.17(-0.47–0.3) | -2.67(-3.63–-1.71) |  | 17.47(9.45–25.69) | -0.13(-0.4–0.26) | -2.12(-2.87–-1.35) |
| Equatorial Guinea | 0.97(0.6–1.5) | 0.06(-0.04–0.19) | 0.8(0.33–1.28) |  | 17.83(8.81–32.96) | -0.15(-0.59–0.51) | -1.92(-2.66–-1.19) |  | 18.81(9.74–34.17) | -0.14(-0.58–0.49) | -1.8(-2.52–-1.07) |
| Eritrea | 0.93(0.58–1.41) | 0.1(-0.01–0.23) | 1.36(0.96–1.77) |  | 38.26(16.73–82.02) | 0.09(-0.34–0.94) | 1.18(0.85–1.51) |  | 39.19(17.61–83.03) | 0.09(-0.33–0.9) | 1.18(0.85–1.52) |
| Estonia | 2.31(1.5–3.37) | -0.02(-0.17–0.16) | -0.14(-0.64–0.36) |  | 17.46(12.02–27.51) | -0.17(-0.36–0.06) | -2.34(-3.52–-1.16) |  | 19.78(14.23–29.85) | -0.16(-0.33–0.05) | -2.1(-3.18–-1.01) |
| Eswatini | 1.91(1.19–2.85) | 0.09(-0.04–0.24) | 1.19(0.64–1.75) |  | 33.76(20.6–52.65) | 0.12(-0.35–0.82) | 1.52(1.09–1.96) |  | 35.67(22.59–53.99) | 0.12(-0.33–0.75) | 1.51(1.07–1.94) |
| Ethiopia | 0.67(0.42–1.03) | 0.25(0.17–0.33) | 2.91(2.08–3.74) |  | 27.41(13.87–55.37) | -0.01(-0.35–0.59) | 0.14(-0.3–0.58) |  | 28.08(14.42–55.99) | 0(-0.34–0.57) | 0.2(-0.24–0.64) |
| Fiji | 1.39(0.85–2.11) | 0.09(-0.01–0.2) | 1.07(1.01–1.12) |  | 53.42(34.24–83.51) | 0.15(-0.24–0.73) | 1.11(0.15–2.08) |  | 54.8(35.69–85.07) | 0.15(-0.23–0.7) | 1.11(0.17–2.05) |
| Finland | 6.61(4.4–9.6) | -0.02(-0.2–0.19) | 0.08(-0.48–0.65) |  | 17.82(11.98–20.91) | 0.12(-0.04–0.28) | 1.21(0.24–2.19) |  | 24.43(18.22–28.82) | 0.08(-0.05–0.2) | 0.89(0.24–1.55) |
| France | 5.7(3.72–8.34) | 0.12(-0.02–0.3) | 1.42(0.97–1.87) |  | 17.26(11.7–20.63) | 0.01(-0.13–0.15) | 0.22(-0.09–0.53) |  | 22.96(17.48–27.09) | 0.03(-0.08–0.14) | 0.5(0.32–0.69) |
| Gabon | 1.08(0.68–1.64) | 0.07(-0.04–0.2) | 0.91(0.53–1.29) |  | 22.92(12.87–38.18) | -0.16(-0.52–0.53) | -1.66(-2.4–-0.92) |  | 24(13.78–38.98) | -0.15(-0.51–0.51) | -1.56(-2.28–-0.83) |
| Gambia | 1.12(0.71–1.68) | 0.05(-0.06–0.17) | 0.65(0.3–0.99) |  | 31.28(15.23–57.59) | 0.02(-0.41–0.77) | 1.22(0.05–2.41) |  | 32.4(16.41–58.65) | 0.02(-0.4–0.73) | 1.2(0.06–2.36) |
| Georgia | 1.97(1.24–2.99) | 0.11(-0.02–0.28) | 1.5(0.69–2.32) |  | 14.69(8.44–21.34) | 0.12(-0.23–0.62) | 0.93(-0.74–2.63) |  | 16.66(10.19–23.38) | 0.12(-0.19–0.55) | 0.99(-0.44–2.44) |
| Germany | 5.56(3.62–8.12) | 0.12(-0.02–0.31) | 1.28(0.83–1.73) |  | 20.93(16.02–24.56) | -0.05(-0.19–0.08) | -0.46(-0.66–-0.25) |  | 26.49(21.24–30.65) | -0.02(-0.13–0.09) | -0.12(-0.26–0.02) |
| Ghana | 1.13(0.7–1.71) | 0.02(-0.08–0.14) | 0.24(0.08–0.4) |  | 39.14(18.84–79.31) | 0.09(-0.36–0.89) | 1.52(0.41–2.64) |  | 40.27(19.89–80.08) | 0.09(-0.36–0.86) | 1.48(0.4–2.57) |
| Greece | 6.66(4.22–9.87) | 0.09(-0.12–0.32) | 1.03(0.84–1.23) |  | 29.13(21.41–41.94) | 0.86(0.45–1.36) | 7.71(5.24–10.24) |  | 35.79(27.61–48.4) | 0.64(0.34–1.02) | 6.19(4.27–8.14) |
| Greenland | 4.05(2.51–6.06) | 0.05(-0.07–0.2) | 0.68(0.16–1.21) |  | 14.68(9.59–24.44) | -0.07(-0.37–0.37) | -0.64(-1.37–0.09) |  | 18.74(13.38–28.83) | -0.05(-0.31–0.3) | -0.37(-1.04–0.31) |
| Grenada | 2.94(1.86–4.32) | 0.12(-0.01–0.26) | 1.43(1.05–1.81) |  | 17.53(12.47–23.18) | -0.08(-0.31–0.19) | -1.23(-1.85–-0.61) |  | 20.48(15.23–26.32) | -0.05(-0.27–0.19) | -0.89(-1.38–-0.39) |
| Guam | 0.87(0.54–1.34) | 0.12(0–0.27) | 1.43(1.15–1.71) |  | 10.48(6.86–15.17) | -0.16(-0.45–0.25) | -2.58(-4.26–-0.86) |  | 11.35(7.6–15.99) | -0.15(-0.41–0.25) | -2.32(-3.89–-0.73) |
| Guatemala | 2.71(1.68–4.16) | 0.07(-0.13–0.27) | 0.99(0.06–1.93) |  | 38.62(20.71–56.53) | -0.1(-0.39–0.3) | -1.62(-2.63–-0.61) |  | 41.33(23.23–58.9) | -0.09(-0.36–0.29) | -1.47(-2.39–-0.55) |
| Guinea | 1.02(0.64–1.54) | 0.05(-0.07–0.15) | 0.58(0.35–0.81) |  | 40.55(14.22–105.93) | -0.02(-0.43–0.79) | 0.15(-0.26–0.57) |  | 41.57(15.24–107.04) | -0.02(-0.43–0.75) | 0.16(-0.24–0.57) |
| Guinea-Bissau | 1.38(0.88–2.06) | 0.03(-0.07–0.14) | 0.34(0.21–0.46) |  | 42.6(15.8–102.15) | -0.14(-0.49–0.56) | -0.91(-1.98–0.17) |  | 43.98(17.07–103.44) | -0.14(-0.48–0.53) | -0.87(-1.91–0.18) |
| Guyana | 2.53(1.56–3.77) | 0.14(0–0.44) | 1.78(1.01–2.54) |  | 8.68(5.21–12.43) | -0.09(-0.38–0.29) | -1.07(-1.64–-0.51) |  | 11.2(7.59–15.12) | -0.04(-0.29–0.26) | -0.49(-0.84–-0.15) |
| Haiti | 3.68(2.3–5.68) | 0.14(-0.01–0.31) | 1.68(1.09–2.27) |  | 76.7(18.78–161.47) | 0.08(-0.29–0.54) | 0.72(0.46–0.97) |  | 80.39(22.55–165.2) | 0.08(-0.27–0.52) | 0.76(0.54–0.98) |
| Honduras | 2.56(1.55–3.88) | 0.16(-0.03–0.46) | 1.86(1.04–2.69) |  | 13.92(7.14–23.62) | -0.19(-0.48–0.26) | -2.17(-2.64–-1.71) |  | 16.47(9.59–26.41) | -0.15(-0.42–0.26) | -1.64(-2.13–-1.15) |
| Hungary | 1.89(1.2–2.78) | 0.16(-0.09–0.5) | 1.54(1.32–1.77) |  | 24.86(16.88–33.72) | -0.13(-0.35–0.12) | -1.47(-2.05–-0.89) |  | 26.75(18.81–35.73) | -0.12(-0.32–0.13) | -1.29(-1.84–-0.73) |
| Iceland | 6.23(3.94–9.25) | 0.09(-0.05–0.25) | 1.14(0.75–1.53) |  | 22.69(16.09–31.78) | -0.08(-0.32–0.29) | -0.97(-1.89–-0.05) |  | 28.92(21.75–38.11) | -0.05(-0.25–0.26) | -0.55(-1.25–0.15) |
| India | 0.93(0.59–1.41) | 0.2(0.14–0.26) | 2.27(1.58–2.97) |  | 12.56(7.4–19.05) | -0.08(-0.35–0.23) | -1.05(-1.36–-0.75) |  | 13.49(8.26–19.84) | -0.06(-0.33–0.23) | -0.85(-1.12–-0.59) |
| Indonesia | 2.76(1.73–4.15) | 0.19(0.11–0.33) | 2.21(1.44–2.97) |  | 14.41(10.44–19.67) | -0.02(-0.29–0.35) | -0.3(-0.47–-0.12) |  | 17.17(12.96–22.69) | 0(-0.24–0.32) | 0.07(-0.04–0.18) |
| Iran (Islamic Republic of) | 2.81(1.78–4.21) | 0.05(-0.02–0.12) | 1.46(0.21–2.74) |  | 33.82(24.33–44.42) | -0.23(-0.49–0.07) | -3.43(-4.37–-2.49) |  | 36.63(26.97–47.15) | -0.21(-0.46–0.07) | -3.1(-3.95–-2.24) |
| Iraq | 2.81(1.66–4.34) | 0.22(0.06–0.6) | 2.64(1.58–3.72) |  | 12.73(8.62–18.28) | -0.23(-0.51–0.1) | -3.18(-3.81–-2.54) |  | 15.54(11.03–21.29) | -0.18(-0.44–0.13) | -2.35(-2.78–-1.91) |
| Ireland | 8.11(5.17–12.13) | 0.08(-0.14–0.33) | 1.02(0.33–1.71) |  | 33.53(26.53–44.14) | 0.11(-0.12–0.39) | 0.85(0.26–1.45) |  | 41.64(33.96–52.44) | 0.11(-0.08–0.33) | 0.88(0.44–1.33) |
| Israel | 5.47(3.6–7.85) | 0.09(-0.05–0.26) | 1.09(0.72–1.47) |  | 16.17(12.86–21.03) | -0.19(-0.34–0.01) | -2.53(-3.5–-1.55) |  | 21.65(17.66–26.68) | -0.13(-0.26–0.02) | -1.71(-2.54–-0.87) |
| Italy | 4.33(2.86–6.29) | 0.06(-0.01–0.14) | 0.92(0.08–1.78) |  | 15.69(11.69–18.88) | 0(-0.14–0.13) | 0.09(-0.16–0.34) |  | 20.02(15.86–23.37) | 0.02(-0.1–0.12) | 0.26(-0.03–0.55) |
| Jamaica | 2.69(1.74–4.03) | 0.13(0.01–0.26) | 1.49(1.21–1.78) |  | 21.99(14.55–31.6) | -0.09(-0.36–0.29) | -1.49(-2.05–-0.94) |  | 24.68(17.02–34.47) | -0.07(-0.32–0.27) | -1.2(-1.7–-0.7) |
| Japan | 4.99(3.12–7.39) | -0.06(-0.11–0.01) | 0.24(-0.91–1.41) |  | 6.12(4.03–7.68) | -0.02(-0.19–0.12) | -0.29(-0.51–-0.08) |  | 11.11(8.31–13.82) | -0.04(-0.12–0.05) | -0.04(-0.56–0.47) |
| Jordan | 2.91(1.85–4.39) | 0.13(-0.01–0.28) | 1.66(1.13–2.19) |  | 48.99(35.22–68.81) | -0.1(-0.34–0.23) | -0.76(-1.47–-0.04) |  | 51.9(38.16–71.43) | -0.09(-0.31–0.22) | -0.64(-1.34–0.07) |
| Kazakhstan | 1.73(1.05–2.64) | 0.09(-0.02–0.23) | 1.27(0.59–1.95) |  | 8.13(4.38–12.53) | -0.15(-0.45–0.28) | -2.04(-2.42–-1.66) |  | 9.86(6.19–14.43) | -0.11(-0.38–0.24) | -1.52(-1.84–-1.2) |
| Kenya | 0.87(0.55–1.32) | 0.19(0.09–0.33) | 2.43(1.44–3.44) |  | 15.32(10.35–22.41) | 0.01(-0.24–0.31) | -0.35(-0.99–0.29) |  | 16.19(11.39–23.27) | 0.02(-0.23–0.31) | -0.22(-0.8–0.37) |
| Kiribati | 1.27(0.76–1.98) | 0.11(-0.02–0.25) | 1.37(0.74–2.01) |  | 15.65(6.14–30.31) | -0.14(-0.45–0.41) | -1.68(-2.26–-1.09) |  | 16.93(7.38–31.81) | -0.13(-0.43–0.38) | -1.47(-2.06–-0.89) |
| Kuwait | 2.69(1.66–4.08) | 0.16(-0.04–0.34) | 1.79(1.45–2.14) |  | 24.38(17.63–34.7) | 0.47(0.08–1.02) | 4.1(2.91–5.31) |  | 27.07(20.21–37.44) | 0.43(0.09–0.91) | 3.85(2.79–4.92) |
| Kyrgyzstan | 1.63(1–2.44) | 0.03(-0.12–0.17) | 0.58(-0.19–1.35) |  | 6.34(3.44–9.52) | -0.26(-0.51–-0.01) | -3.67(-5.04–-2.29) |  | 7.96(4.96–11.42) | -0.21(-0.41–0) | -2.9(-4.01–-1.77) |
| Lao People's Democratic Republic | 1.54(0.93–2.35) | 0.24(0.1–0.4) | 2.7(2.04–3.36) |  | 12.44(6.29–22.07) | -0.22(-0.58–0.44) | -2.72(-3.08–-2.35) |  | 13.98(7.82–23.66) | -0.18(-0.55–0.42) | -2.23(-2.62–-1.85) |
| Latvia | 2.45(1.53–3.67) | -0.03(-0.17–0.13) | -0.36(-0.64–-0.08) |  | 32.21(21.99–54.63) | -0.24(-0.44–0.01) | -3.39(-5.31–-1.44) |  | 34.66(24.44–56.77) | -0.23(-0.42–0.01) | -3.2(-5.01–-1.35) |
| Lebanon | 2.88(1.76–4.35) | 0.1(-0.03–0.26) | 1.18(0.69–1.68) |  | 22.7(10–39.45) | -0.18(-0.48–0.22) | -1.65(-2.32–-0.97) |  | 25.59(12.55–42.38) | -0.15(-0.44–0.21) | -1.37(-2.01–-0.73) |
| Lesotho | 2.17(1.36–3.24) | 0.09(-0.02–0.23) | 1.3(0.75–1.85) |  | 36.08(22.02–54.66) | 0.18(-0.2–0.75) | 1.56(1.22–1.91) |  | 38.24(24.13–56.57) | 0.17(-0.19–0.69) | 1.55(1.22–1.87) |
| Liberia | 1.01(0.64–1.52) | 0.05(-0.05–0.17) | 0.63(0.36–0.91) |  | 27.82(12.58–64.72) | 0.02(-0.38–0.8) | -0.59(-1.99–0.82) |  | 28.83(13.67–66.23) | 0.02(-0.37–0.76) | -0.55(-1.9–0.81) |
| Libya | 3.49(2.21–5.27) | 0.11(-0.04–0.28) | 1.42(0.82–2.02) |  | 65.17(40.12–101.1) | 0.1(-0.26–0.64) | 1.34(1.01–1.67) |  | 68.66(43.95–104.29) | 0.1(-0.24–0.61) | 1.34(1.01–1.68) |
| Lithuania | 2.49(1.57–3.69) | 0.11(-0.06–0.3) | 1.48(0.81–2.14) |  | 16.2(11.48–22.44) | -0.01(-0.25–0.27) | 0.61(-0.32–1.53) |  | 18.68(13.92–25.07) | 0(-0.21–0.25) | 0.72(-0.1–1.54) |
| Luxembourg | 5.65(3.6–8.24) | 0.13(-0.01–0.28) | 1.63(1.07–2.19) |  | 5.74(3.83–8.21) | 0.06(-0.21–0.47) | 0.94(0.47–1.41) |  | 11.4(8.47–14.72) | 0.09(-0.07–0.32) | 1.27(1–1.55) |
| Madagascar | 0.93(0.59–1.42) | 0.08(-0.04–0.25) | 1(0.51–1.49) |  | 22.65(11.34–44.7) | -0.08(-0.43–0.57) | -0.86(-1.17–-0.56) |  | 23.58(12.39–45.6) | -0.08(-0.42–0.54) | -0.8(-1.08–-0.51) |
| Malawi | 1.1(0.68–1.63) | 0.08(-0.04–0.22) | 0.94(0.58–1.3) |  | 30.54(15.09–59.28) | -0.03(-0.44–0.77) | -1(-1.91–-0.09) |  | 31.63(16.18–60.1) | -0.03(-0.43–0.74) | -0.94(-1.81–-0.06) |
| Malaysia | 1.19(0.72–1.84) | 0.19(0.02–0.41) | 2.14(1.48–2.81) |  | 4.7(3.17–6.85) | -0.06(-0.36–0.36) | -0.16(-1.37–1.06) |  | 5.89(4.22–8.15) | -0.02(-0.27–0.34) | 0.26(-0.73–1.26) |
| Maldives | 1.27(0.77–1.92) | 0.05(-0.12–0.23) | 0.56(0.23–0.89) |  | 65.37(38.98–100.23) | 0.12(-0.32–0.79) | 1.86(1.29–2.44) |  | 66.65(40.01–101.82) | 0.12(-0.32–0.77) | 1.84(1.28–2.4) |
| Mali | 1.03(0.65–1.55) | 0.09(-0.02–0.2) | 1.19(0.78–1.6) |  | 49.9(13.79–151.87) | 0.06(-0.38–0.97) | 1.17(0.3–2.05) |  | 50.93(14.72–152.91) | 0.06(-0.38–0.91) | 1.17(0.31–2.04) |
| Malta | 8.62(5.5–12.59) | 0.04(-0.12–0.22) | 0.78(0.22–1.34) |  | 30.61(22.68–41.77) | 0.08(-0.2–0.48) | 0.87(0.3–1.44) |  | 39.23(30.1–49.91) | 0.07(-0.16–0.37) | 0.85(0.44–1.26) |
| Marshall Islands | 1.3(0.78–2.04) | 0.15(0.03–0.33) | 1.82(1.27–2.38) |  | 11.91(7.32–17.78) | -0.01(-0.34–0.48) | -0.16(-0.4–0.09) |  | 13.21(8.66–19.07) | 0(-0.31–0.45) | 0.02(-0.24–0.29) |
| Mauritania | 1.12(0.71–1.66) | 0.06(-0.05–0.16) | 0.61(0.4–0.82) |  | 28.07(12.03–57.69) | -0.17(-0.55–0.49) | -2.32(-3.43–-1.2) |  | 29.2(13.29–58.95) | -0.16(-0.53–0.47) | -2.23(-3.29–-1.15) |
| Mauritius | 1.35(0.83–2.1) | 0.21(0.04–0.39) | 2.38(1.72–3.04) |  | 7.84(5.39–10.82) | 0.52(0.1–1.04) | 6.68(4.18–9.24) |  | 9.18(6.62–12.29) | 0.46(0.11–0.89) | 5.99(3.88–8.15) |
| Mexico | 2.03(1.3–3.08) | 0.01(-0.06–0.08) | 0.8(-0.21–1.82) |  | 29.09(20.86–37.26) | -0.05(-0.27–0.22) | -0.73(-0.95–-0.51) |  | 31.13(22.83–39.48) | -0.05(-0.25–0.2) | -0.63(-0.83–-0.43) |
| Micronesia (Federated States of) | 1.27(0.77–1.95) | 0.12(0–0.27) | 1.54(1–2.08) |  | 11.97(7.01–18.12) | -0.08(-0.39–0.41) | -0.8(-0.98–-0.62) |  | 13.24(8.27–19.29) | -0.06(-0.36–0.37) | -0.6(-0.77–-0.43) |
| Monaco | 6.15(3.87–9.13) | 0.11(-0.02–0.26) | 1.35(0.91–1.78) |  | 3.41(2.34–4.76) | -0.04(-0.3–0.3) | -0.25(-0.58–0.08) |  | 9.56(7.04–12.78) | 0.05(-0.09–0.2) | 0.75(0.36–1.13) |
| Mongolia | 1.8(1.1–2.74) | 0.13(-0.02–0.41) | 1.6(0.77–2.43) |  | 11.23(6.83–20.02) | -0.26(-0.53–0.17) | -3.62(-4.54–-2.68) |  | 13.03(8.5–21.63) | -0.22(-0.47–0.16) | -3.03(-3.91–-2.13) |
| Montenegro | 1.87(1.19–2.73) | 0.15(-0.03–0.39) | 1.64(1.46–1.82) |  | 22.39(14.02–33.07) | -0.24(-0.48–0.07) | -2.78(-3.29–-2.26) |  | 24.25(15.82–34.98) | -0.22(-0.45–0.07) | -2.5(-2.99–-2) |
| Morocco | 3.39(2.07–5.21) | 0.19(0.04–0.38) | 2.2(1.49–2.92) |  | 33.44(14.83–64.81) | -0.01(-0.4–0.59) | 0.21(-0.09–0.51) |  | 36.83(18.21–68.72) | 0.01(-0.36–0.56) | 0.38(0.09–0.66) |
| Mozambique | 1(0.62–1.54) | 0.1(-0.03–0.28) | 1.1(0.89–1.31) |  | 38.39(16.61–81.97) | -0.02(-0.46–0.84) | -0.58(-1.21–0.04) |  | 39.4(17.71–82.76) | -0.02(-0.45–0.82) | -0.54(-1.15–0.07) |
| Myanmar | 1.71(1.07–2.58) | 0.2(0.08–0.31) | 2.19(1.62–2.77) |  | 20.44(10.53–40.46) | -0.15(-0.55–0.67) | -1.65(-2.14–-1.15) |  | 22.15(12.27–41.99) | -0.13(-0.53–0.62) | -1.4(-1.9–-0.89) |
| Namibia | 1.75(1.11–2.69) | 0.05(-0.06–0.19) | 0.84(0.18–1.5) |  | 32.41(19.04–51.54) | -0.01(-0.38–0.58) | 0.04(-0.36–0.44) |  | 34.16(20.71–53.41) | -0.01(-0.36–0.54) | 0.08(-0.29–0.44) |
| Nauru | 1.16(0.7–1.79) | 0.17(0.05–0.34) | 1.91(1.26–2.55) |  | 15.57(9–25.77) | -0.18(-0.55–0.49) | -2.36(-2.77–-1.96) |  | 16.73(10.17–26.99) | -0.16(-0.53–0.48) | -2.12(-2.54–-1.7) |
| Nepal | 0.92(0.57–1.38) | 0.15(0.03–0.31) | 1.81(1.09–2.54) |  | 10.3(5.59–16.86) | -0.17(-0.5–0.46) | -1.95(-2.23–-1.66) |  | 11.22(6.49–17.76) | -0.15(-0.47–0.43) | -1.68(-1.95–-1.42) |
| Netherlands | 6.26(4.12–8.92) | 0.02(-0.13–0.16) | 0.24(0.16–0.33) |  | 27.51(17.52–33.9) | -0.11(-0.25–0.02) | -1.19(-1.31–-1.07) |  | 33.77(23.33–40.21) | -0.09(-0.21–0.02) | -0.94(-1.04–-0.84) |
| New Zealand | 5.09(3.28–7.44) | 0.06(-0.07–0.19) | 0.76(0.33–1.18) |  | 25.68(17.03–33.83) | -0.15(-0.37–0.06) | -1.79(-2.28–-1.3) |  | 30.77(22.01–38.97) | -0.12(-0.31–0.07) | -1.41(-1.87–-0.95) |
| Nicaragua | 2.42(1.49–3.63) | 0.09(-0.08–0.28) | 1.15(0.55–1.75) |  | 11.52(8.35–15.7) | -0.18(-0.4–0.11) | -1.73(-2.95–-0.49) |  | 13.94(10.7–18.1) | -0.14(-0.34–0.12) | -1.28(-2.41–-0.14) |
| Niger | 0.94(0.59–1.4) | 0.13(0.01–0.25) | 1.59(1.28–1.89) |  | 35.42(7.72–104.74) | 0.01(-0.42–0.84) | 0.55(-0.55–1.67) |  | 36.36(8.75–105.41) | 0.02(-0.41–0.79) | 0.58(-0.5–1.66) |
| Nigeria | 0.86(0.54–1.31) | 0.14(0.09–0.2) | 1.83(1.09–2.57) |  | 46.08(15.24–129.54) | -0.06(-0.31–0.47) | -0.52(-1.11–0.07) |  | 46.94(16.02–130.36) | -0.06(-0.31–0.46) | -0.49(-1.06–0.09) |
| Niue | 1.09(0.68–1.65) | 0.12(0–0.26) | 1.42(0.98–1.86) |  | 17.93(10.11–29.48) | 0.02(-0.34–0.57) | 0.31(0.09–0.52) |  | 19.02(11.1–30.7) | 0.03(-0.32–0.55) | 0.37(0.15–0.58) |
| North Macedonia | 1.73(1.08–2.6) | 0.11(-0.03–0.29) | 1.39(0.96–1.82) |  | 22.89(13.77–34.9) | -0.23(-0.52–0.29) | -1.78(-3.16–-0.38) |  | 24.62(15.48–36.69) | -0.22(-0.49–0.27) | -1.59(-2.9–-0.27) |
| Northern Mariana Islands | 1.02(0.63–1.56) | 0.1(-0.04–0.25) | 1.12(0.87–1.36) |  | 5.13(3.02–7.65) | -0.51(-0.73–-0.2) | -8.7(-10.36–-7) |  | 6.15(4.03–8.72) | -0.46(-0.68–-0.17) | -7.65(-9.12–-6.15) |
| Norway | 5.31(3.54–7.64) | 0(-0.09–0.11) | 0.28(-0.22–0.79) |  | 18.07(11.6–22.16) | -0.1(-0.24–0.03) | -1.21(-1.39–-1.03) |  | 23.38(16.59–27.89) | -0.08(-0.18–0.02) | -0.88(-1.08–-0.69) |
| Oman | 3.36(2.08–5.04) | 0.16(0.02–0.31) | 1.93(1.5–2.37) |  | 34.82(20.87–54.6) | 0.2(-0.16–0.72) | 2.27(1.53–3.02) |  | 38.18(23.75–58.31) | 0.19(-0.14–0.66) | 2.24(1.56–2.93) |
| Pakistan | 1.21(0.74–1.85) | 0.04(-0.05–0.14) | 1.3(0.29–2.31) |  | 16.71(8.58–28.11) | 0(-0.29–0.44) | 0.18(-0.31–0.68) |  | 17.92(9.67–28.97) | 0(-0.27–0.4) | 0.26(-0.22–0.74) |
| Palau | 1.11(0.68–1.68) | 0.17(0.06–0.34) | 2.04(1.32–2.77) |  | 3.45(2.12–5.06) | 0.14(-0.2–0.68) | 1.73(1.48–1.97) |  | 4.56(3.19–6.34) | 0.15(-0.13–0.54) | 1.8(1.59–2.02) |
| Palestine | 2.78(1.69–4.19) | 0.14(0–0.29) | 1.68(1.01–2.35) |  | 38.96(28.39–52.95) | -0.12(-0.39–0.27) | -1.48(-1.74–-1.22) |  | 41.74(31.07–55.51) | -0.1(-0.37–0.26) | -1.3(-1.53–-1.05) |
| Panama | 2.04(1.29–3.05) | 0.06(-0.1–0.26) | 0.9(0.41–1.38) |  | 18(12.19–25.66) | 0.12(-0.22–0.59) | 0.96(0.19–1.74) |  | 20.03(14.15–27.97) | 0.12(-0.19–0.53) | 0.95(0.29–1.63) |
| Papua New Guinea | 1.29(0.78–1.97) | 0.15(0.02–0.27) | 1.79(1.1–2.48) |  | 26.94(8.9–77.72) | -0.01(-0.36–0.73) | -0.12(-0.4–0.17) |  | 28.23(10.34–78.92) | 0(-0.35–0.67) | -0.04(-0.33–0.27) |
| Paraguay | 2.56(1.59–3.91) | 0.1(-0.03–0.27) | 1.29(0.79–1.79) |  | 79.7(50.7–119.87) | 0.04(-0.35–0.7) | 0.41(-0.15–0.98) |  | 82.26(53.98–122.98) | 0.05(-0.35–0.68) | 0.44(-0.1–0.98) |
| Peru | 1.47(0.91–2.24) | 0.09(-0.03–0.22) | 1.06(0.55–1.58) |  | 15.27(7.12–25.17) | -0.26(-0.58–0.22) | -3.35(-5.27–-1.39) |  | 16.74(8.58–26.62) | -0.24(-0.55–0.22) | -3.02(-4.84–-1.16) |
| Philippines | 1.36(0.86–2.03) | 0.27(0.16–0.41) | 3.19(1.63–4.77) |  | 17.48(11.73–22.24) | 0.01(-0.24–0.25) | 0.01(-0.9–0.93) |  | 18.84(13.1–23.75) | 0.02(-0.2–0.25) | 0.21(-0.58–1) |
| Poland | 2.82(1.79–4.15) | 0.58(0.42–0.87) | 5.41(5.13–5.7) |  | 6.05(4.18–8.09) | 0.07(-0.16–0.32) | 0.78(0.37–1.19) |  | 8.87(6.58–11.17) | 0.19(0–0.39) | 2.04(1.77–2.31) |
| Portugal | 5.67(3.68–8.39) | 0.11(-0.04–0.27) | 1.45(0.99–1.91) |  | 19.09(14.9–25.22) | 0.14(-0.08–0.39) | 1.47(0.78–2.17) |  | 24.76(19.95–31.19) | 0.13(-0.04–0.33) | 1.46(0.98–1.95) |
| Puerto Rico | 2.64(1.7–3.9) | 0.01(-0.17–0.24) | 0.66(0–1.32) |  | 25.67(19.29–34.09) | -0.04(-0.27–0.27) | -0.86(-1.79–0.09) |  | 28.31(21.94–36.99) | -0.03(-0.25–0.25) | -0.72(-1.56–0.14) |
| Qatar | 3.03(1.92–4.48) | 0.08(-0.06–0.24) | 0.96(0.67–1.25) |  | 26.34(18.08–37.09) | 0.09(-0.23–0.54) | 0.69(-0.36–1.75) |  | 29.38(21.1–39.94) | 0.09(-0.2–0.48) | 0.72(-0.24–1.69) |
| Republic of Korea | 5.4(3.35–7.92) | 0.08(-0.08–0.25) | 1.02(0.59–1.46) |  | 7.36(5.11–9.92) | -0.03(-0.29–0.27) | 0.06(-0.76–0.88) |  | 12.75(9.7–16.83) | 0.01(-0.17–0.21) | 0.45(-0.19–1.1) |
| Republic of Moldova | 2.38(1.48–3.56) | 0.15(-0.04–0.49) | 1.97(1.01–2.93) |  | 6.76(5.12–8.97) | -0.21(-0.38–0) | -2.88(-4.17–-1.58) |  | 9.14(7.2–11.6) | -0.14(-0.3–0.04) | -1.77(-2.89–-0.64) |
| Romania | 1.72(1.08–2.58) | 0.12(-0.03–0.37) | 1.43(0.98–1.88) |  | 3.63(2.7–4.76) | -0.12(-0.36–0.18) | -1.45(-2.08–-0.81) |  | 5.35(4.15–6.88) | -0.06(-0.24–0.17) | -0.6(-1.13–-0.06) |
| Russian Federation | 2.27(1.42–3.45) | -0.09(-0.25–-0.01) | -0.45(-1.29–0.39) |  | 11.57(7.46–15.81) | -0.06(-0.23–0.16) | -1.16(-2.81–0.53) |  | 13.83(9.65–18.03) | -0.06(-0.21–0.13) | -1.02(-2.38–0.36) |
| Rwanda | 1.07(0.67–1.63) | 0.06(-0.06–0.18) | 0.74(0.3–1.17) |  | 40.82(17.21–89.16) | -0.01(-0.41–0.83) | 0.16(-0.42–0.75) |  | 41.89(18.34–90.56) | 0(-0.4–0.79) | 0.18(-0.39–0.75) |
| Saint Kitts and Nevis | 2.83(1.8–4.21) | 0.13(0–0.27) | 1.51(1.13–1.88) |  | 17(11.17–23.9) | -0.05(-0.31–0.24) | 0.33(-1.24–1.92) |  | 19.83(13.89–26.9) | -0.03(-0.26–0.22) | 0.48(-0.88–1.86) |
| Saint Lucia | 2.7(1.68–4.17) | 0.12(-0.01–0.28) | 1.5(0.97–2.04) |  | 21.45(15.21–29.35) | 0.08(-0.16–0.4) | 0.77(0.45–1.1) |  | 24.14(18.06–32.13) | 0.09(-0.14–0.38) | 0.85(0.59–1.11) |
| Saint Vincent and the Grenadines | 2.76(1.78–4.14) | 0.1(-0.05–0.26) | 1.22(0.9–1.55) |  | 23.54(16.5–32.89) | 0.07(-0.2–0.45) | 0.94(0.6–1.27) |  | 26.3(19.18–36.09) | 0.08(-0.18–0.4) | 0.97(0.68–1.25) |
| Samoa | 1.1(0.68–1.67) | 0.12(-0.02–0.31) | 1.44(0.93–1.95) |  | 8.71(5.13–13.53) | -0.18(-0.48–0.24) | -1.67(-2.51–-0.82) |  | 9.81(6.2–14.68) | -0.15(-0.43–0.23) | -1.37(-2.13–-0.61) |
| San Marino | 6.63(4.21–9.74) | 0.1(-0.01–0.25) | 1.33(0.89–1.78) |  | 2.57(1.5–3.94) | 0.01(-0.31–0.45) | 0.09(-0.41–0.59) |  | 9.2(6.51–12.63) | 0.08(-0.07–0.23) | 0.97(0.73–1.21) |
| Sao Tome and Principe | 1.13(0.71–1.72) | 0.02(-0.08–0.12) | 0.2(0.14–0.27) |  | 38.22(20.84–64.72) | 0.08(-0.34–0.82) | 3.24(0.47–6.09) |  | 39.36(21.94–65.67) | 0.08(-0.33–0.78) | 3.14(0.46–5.9) |
| Saudi Arabia | 2.51(1.56–3.8) | -0.11(-0.24–0.04) | -0.65(-1.76–0.47) |  | 36.04(20.89–55.23) | 0.07(-0.24–0.55) | 1.07(0.73–1.43) |  | 38.56(23.3–57.69) | 0.06(-0.23–0.5) | 0.95(0.58–1.32) |
| Senegal | 1.22(0.77–1.83) | 0.05(-0.06–0.16) | 0.57(0.47–0.68) |  | 42.69(17.77–85.92) | 0.05(-0.37–0.84) | 1.37(-0.16–2.91) |  | 43.91(19.19–87.12) | 0.05(-0.37–0.8) | 1.34(-0.14–2.85) |
| Serbia | 1.61(1.03–2.36) | 0(-0.13–0.14) | 0.05(-0.48–0.58) |  | 9.97(6.85–15.02) | -0.32(-0.53–-0.03) | -3.65(-6.12–-1.11) |  | 11.58(8.45–16.49) | -0.29(-0.48–-0.03) | -3.22(-5.45–-0.93) |
| Seychelles | 1.41(0.87–2.2) | 0.14(0.02–0.27) | 1.52(1.2–1.84) |  | 14.14(8.35–21.18) | 0.08(-0.25–0.53) | 0.94(0.47–1.41) |  | 15.55(9.63–22.64) | 0.09(-0.22–0.5) | 0.99(0.58–1.4) |
| Sierra Leone | 1.07(0.67–1.64) | 0.04(-0.06–0.15) | 0.51(0.35–0.67) |  | 53.43(16.02–153.69) | -0.06(-0.46–1.01) | -0.85(-1.22–-0.47) |  | 54.5(17.36–155.17) | -0.06(-0.46–0.96) | -0.82(-1.19–-0.45) |
| Singapore | 5.65(3.49–8.56) | 0.1(-0.06–0.29) | 1.28(0.8–1.76) |  | 3.94(2.77–5.68) | 0.16(-0.16–0.58) | 2.8(1.21–4.42) |  | 9.59(7.13–12.82) | 0.12(-0.04–0.31) | 1.87(1.02–2.73) |
| Slovakia | 1.62(1.02–2.42) | 0.16(-0.1–0.54) | 1.81(1.62–2.01) |  | 13.79(9.86–19.34) | 0.02(-0.25–0.41) | 0.19(-0.45–0.84) |  | 15.41(11.49–21.03) | 0.04(-0.21–0.36) | 0.35(-0.24–0.94) |
| Slovenia | 1.65(1.03–2.5) | 0.1(-0.05–0.31) | 0.79(0.43–1.16) |  | 17.04(12.37–24.48) | -0.18(-0.35–0.02) | -1.54(-2.82–-0.25) |  | 18.69(13.76–25.94) | -0.16(-0.33–0.02) | -1.36(-2.55–-0.15) |
| Solomon Islands | 1.38(0.83–2.14) | 0.14(0.01–0.27) | 1.62(1.09–2.16) |  | 16.86(8.01–30.8) | 0(-0.32–0.56) | 0.2(-0.26–0.66) |  | 18.24(9.35–32.05) | 0.01(-0.3–0.51) | 0.3(-0.11–0.7) |
| Somalia | 0.91(0.56–1.38) | 0.17(0.02–0.32) | 2.07(1.43–2.71) |  | 42.54(12.8–121.01) | 0.17(-0.27–0.96) | 1.5(1.03–1.97) |  | 43.45(13.76–121.61) | 0.17(-0.27–0.94) | 1.51(1.06–1.97) |
| South Africa | 1.55(0.99–2.36) | 0.11(0.01–0.23) | 1.76(0.5–3.02) |  | 32.81(23.65–42.78) | 0.18(-0.17–0.58) | 1.81(0.95–2.67) |  | 34.36(25.23–44.47) | 0.17(-0.14–0.56) | 1.8(1.02–2.59) |
| South Sudan | 0.79(0.49–1.22) | 0.13(0–0.28) | 1.68(1.05–2.33) |  | 41.02(12.4–110.81) | 0.2(-0.27–1) | 3.12(1.25–5.02) |  | 41.81(13.27–111.4) | 0.2(-0.27–0.99) | 3.09(1.26–4.96) |
| Spain | 4.92(3.22–7.13) | 0.12(-0.07–0.34) | 1.3(0.88–1.73) |  | 18.43(15.26–22.62) | 0.14(-0.03–0.33) | 1.47(0.86–2.08) |  | 23.35(19.63–27.65) | 0.14(-0.01–0.3) | 1.44(0.87–2) |
| Sri Lanka | 1.28(0.78–1.99) | 0.12(-0.02–0.3) | 1.46(0.95–1.96) |  | 8.37(4.81–13.69) | 0(-0.36–0.61) | 0.13(-0.48–0.74) |  | 9.65(5.97–15.04) | 0.02(-0.31–0.55) | 0.3(-0.19–0.78) |
| Sudan | 2.95(1.73–4.59) | 0.21(0.08–0.37) | 2.5(1.69–3.31) |  | 55.65(28.68–98.29) | -0.11(-0.48–0.56) | -1.44(-1.86–-1.03) |  | 58.59(31.58–101.44) | -0.1(-0.46–0.54) | -1.28(-1.64–-0.91) |
| Suriname | 2.77(1.74–4.18) | 0.12(0.01–0.27) | 1.55(0.86–2.25) |  | 19.39(13.63–28.9) | -0.03(-0.31–0.36) | -0.33(-0.65–0) |  | 22.16(16.13–31.86) | -0.01(-0.27–0.32) | -0.11(-0.46–0.25) |
| Sweden | 5.98(4–8.62) | 0.09(-0.03–0.22) | 1.35(0.75–1.96) |  | 20.13(13.52–24.06) | -0.01(-0.19–0.13) | -0.06(-0.75–0.63) |  | 26.12(18.9–30.72) | 0.01(-0.13–0.13) | 0.24(-0.27–0.76) |
| Switzerland | 5.65(3.62–8.41) | 0.05(-0.08–0.2) | 0.66(0.47–0.86) |  | 25.13(18.11–30.31) | -0.05(-0.19–0.12) | -0.68(-1.18–-0.19) |  | 30.78(23.31–36.67) | -0.03(-0.15–0.11) | -0.45(-0.84–-0.06) |
| Syrian Arab Republic | 3.53(2.24–5.4) | 0.13(-0.02–0.29) | 1.57(1–2.15) |  | 49.32(28.1–81.61) | 0.22(-0.21–0.89) | 2.31(1.15–3.49) |  | 52.86(31.3–84.89) | 0.21(-0.19–0.82) | 2.26(1.2–3.34) |
| Taiwan (Province of China) | 1.07(0.66–1.64) | 0.11(-0.01–0.24) | 1.25(1–1.5) |  | 12(8.75–16.9) | 0.31(-0.09–0.91) | 2.89(1.74–4.06) |  | 13.07(9.68–18.02) | 0.29(-0.08–0.82) | 2.75(1.68–3.83) |
| Tajikistan | 1.65(1.03–2.52) | 0.14(-0.01–0.33) | 1.87(0.91–2.83) |  | 14.44(7.33–23.49) | 0.05(-0.39–0.77) | 0.53(-0.45–1.53) |  | 16.09(9.01–25.22) | 0.06(-0.35–0.68) | 0.66(-0.18–1.51) |
| Thailand | 1.29(0.8–1.99) | 0.2(0.02–0.43) | 2.31(1.63–2.98) |  | 7.36(4.52–10.63) | 0.32(-0.15–0.96) | 4.43(2.78–6.11) |  | 8.65(5.73–11.9) | 0.3(-0.1–0.82) | 4.11(2.7–5.53) |
| Timor-Leste | 1.66(1.01–2.51) | 0.21(0.06–0.43) | 2.32(1.7–2.96) |  | 14.61(8.46–23.22) | -0.13(-0.49–0.58) | -1.77(-2.61–-0.93) |  | 16.27(10.04–24.77) | -0.1(-0.45–0.52) | -1.43(-2.14–-0.7) |
| Togo | 1.1(0.68–1.62) | 0.04(-0.05–0.15) | 0.41(0.17–0.66) |  | 33.73(14.22–86.6) | 0(-0.36–0.69) | 0.16(-0.3–0.62) |  | 34.83(15.45–87.58) | 0(-0.35–0.66) | 0.17(-0.28–0.62) |
| Tokelau | 1.13(0.7–1.71) | 0.12(0–0.25) | 1.49(0.92–2.07) |  | 9.95(5.91–15.39) | -0.18(-0.51–0.27) | -2.28(-2.7–-1.86) |  | 11.08(6.94–16.53) | -0.15(-0.47–0.26) | -1.96(-2.3–-1.61) |
| Tonga | 1.13(0.7–1.72) | 0.12(0–0.25) | 1.39(0.91–1.88) |  | 10.72(6.64–17.53) | -0.07(-0.41–0.53) | -0.81(-0.94–-0.68) |  | 11.84(7.61–18.69) | -0.05(-0.38–0.48) | -0.62(-0.71–-0.53) |
| Trinidad and Tobago | 2.97(1.93–4.44) | 0.06(-0.06–0.2) | 0.78(0.46–1.11) |  | 50.47(34.41–71.68) | 0.08(-0.23–0.47) | 1.13(0.24–2.02) |  | 53.45(37.28–74.38) | 0.08(-0.22–0.45) | 1.11(0.26–1.96) |
| Tunisia | 2.67(1.64–3.98) | 0.1(-0.01–0.24) | 1.16(0.81–1.52) |  | 40.95(24.75–60.46) | -0.04(-0.37–0.41) | -0.43(-0.55–-0.31) |  | 43.62(26.96–63.27) | -0.03(-0.35–0.39) | -0.34(-0.47–-0.21) |
| Turkey | 2.36(1.47–3.6) | -0.01(-0.13–0.12) | 0.23(-0.24–0.7) |  | 45.81(32.54–63.38) | -0.31(-0.53–0.03) | -4.1(-4.73–-3.47) |  | 48.17(34.71–65.76) | -0.3(-0.52–0.03) | -3.92(-4.54–-3.31) |
| Turkmenistan | 1.81(1.11–2.7) | 0.07(-0.05–0.19) | 0.93(0.46–1.41) |  | 13.51(5.3–21.21) | -0.23(-0.53–0.2) | -3.25(-3.95–-2.55) |  | 15.32(7.02–23.23) | -0.2(-0.46–0.18) | -2.84(-3.43–-2.25) |
| Tuvalu | 1.14(0.68–1.77) | 0.13(0–0.27) | 1.61(0.93–2.31) |  | 11.67(7.18–18.16) | -0.24(-0.53–0.24) | -2.84(-3.34–-2.35) |  | 12.81(8.22–19.08) | -0.22(-0.51–0.22) | -2.51(-3.03–-2) |
| Uganda | 1(0.64–1.53) | 0.1(-0.02–0.26) | 1.22(0.74–1.7) |  | 23.97(11.52–47.09) | -0.05(-0.44–0.71) | -0.6(-2.5–1.34) |  | 24.98(12.53–48.3) | -0.05(-0.43–0.67) | -0.54(-2.36–1.32) |
| Ukraine | 2.36(1.45–3.54) | 0.05(-0.1–0.27) | 1.26(0.42–2.11) |  | 16.35(12.2–22.16) | 0.38(0.02–0.83) | 4.28(3.1–5.47) |  | 18.71(14.47–24.69) | 0.33(0.02–0.7) | 3.85(2.8–4.91) |
| United Arab Emirates | 3.85(2.41–5.89) | 0.16(0.01–0.34) | 2.01(1.43–2.6) |  | 68.31(35.1–114.49) | 1.15(0.28–3.59) | 9.43(7.83–11.06) |  | 72.16(39.16–118.68) | 1.06(0.28–3.12) | 8.87(7.33–10.43) |
| United Kingdom | 7.35(4.77–10.97) | 0.15(0.09–0.21) | 1.66(0.97–2.37) |  | 16.58(11.77–18.84) | 0.11(0.01–0.19) | 1.71(1.04–2.39) |  | 23.93(18.37–28.18) | 0.12(0.05–0.18) | 1.7(1.25–2.15) |
| United Republic of Tanzania | 1.02(0.63–1.57) | 0.08(-0.03–0.2) | 1.03(0.71–1.35) |  | 41.36(18.32–86.39) | -0.07(-0.45–0.78) | -0.89(-1.53–-0.24) |  | 42.37(19.73–87.48) | -0.07(-0.45–0.76) | -0.85(-1.47–-0.22) |
| United States of America | 3.33(2.16–4.79) | 0.18(0.05–0.31) | 1.64(0.6–2.7) |  | 14.86(12.24–16.73) | 0.03(-0.05–0.1) | 0.53(0.16–0.9) |  | 18.19(15.3–20.33) | 0.05(-0.02–0.12) | 0.72(0.3–1.14) |
| United States Virgin Islands | 2.56(1.65–3.78) | 0.12(-0.01–0.25) | 1.39(1.05–1.73) |  | 18.16(12.92–25.72) | -0.04(-0.29–0.3) | -0.53(-0.87–-0.19) |  | 20.72(15.42–28.67) | -0.03(-0.25–0.27) | -0.31(-0.63–0.02) |
| Uruguay | 4.84(3.04–7.22) | -0.02(-0.15–0.12) | -0.29(-1.12–0.56) |  | 46.61(33.77–60.2) | -0.19(-0.39–0.08) | -2.75(-4.01–-1.47) |  | 51.44(38.54–64.6) | -0.17(-0.36–0.07) | -2.54(-3.75–-1.3) |
| Uzbekistan | 1.59(0.97–2.42) | 0.01(-0.19–0.16) | 0.51(-0.75–1.78) |  | 3.83(2.56–5.25) | -0.18(-0.43–0.1) | -1.69(-3.19–-0.16) |  | 5.42(4.1–7.02) | -0.14(-0.33–0.09) | -1.08(-2.45–0.3) |
| Vanuatu | 1.14(0.69–1.73) | 0.14(0.02–0.28) | 1.73(1.13–2.33) |  | 11.89(6.65–19.51) | -0.1(-0.41–0.44) | -0.88(-1.4–-0.36) |  | 13.03(7.87–20.74) | -0.08(-0.37–0.4) | -0.67(-1.2–-0.15) |
| Venezuela (Bolivarian Republic of) | 2.57(1.64–3.87) | 0.13(-0.05–0.37) | 1.71(1.21–2.21) |  | 38.87(25.73–54.7) | 0.12(-0.19–0.55) | 1.36(0.91–1.81) |  | 41.44(28.19–56.91) | 0.12(-0.17–0.52) | 1.38(0.96–1.81) |
| Viet Nam | 1.2(0.74–1.85) | 0.15(0.01–0.35) | 1.79(1.3–2.27) |  | 8.1(3.94–15.23) | -0.04(-0.39–0.44) | -0.35(-0.63–-0.07) |  | 9.3(5.07–16.58) | -0.02(-0.35–0.4) | -0.1(-0.39–0.19) |
| Yemen | 3.05(1.8–4.82) | 0.18(0.03–0.35) | 2.13(1.36–2.91) |  | 57.19(30.39–109.27) | 0.07(-0.28–0.69) | 0.95(0.76–1.14) |  | 60.24(33.58–112.61) | 0.08(-0.26–0.65) | 1.01(0.81–1.2) |
| Zambia | 1(0.62–1.52) | 0.08(-0.04–0.22) | 1.09(0.7–1.47) |  | 29.37(16.67–51.73) | -0.05(-0.46–0.83) | -0.67(-0.99–-0.35) |  | 30.37(17.83–53.11) | -0.05(-0.45–0.8) | -0.62(-0.93–-0.3) |
| Zimbabwe | 1.92(1.18–2.93) | 0.09(-0.03–0.23) | 1.25(0.69–1.82) |  | 33.03(20.95–50.38) | 0.25(-0.18–0.87) | 2.32(1.83–2.81) |  | 34.96(22.86–52.15) | 0.24(-0.17–0.82) | 2.25(1.8–2.71) |

**Abbreviations:** YLDs, years Lived with Disability; YLLs, years of Life Lost; DALYs, disability-Adjusted Life Years; ASR, age-standardized rate; CI, confidence interval; EAPC, estimated annual percentage change; UI, uncertainty interval.
